# Foot-and-Mouth Disease Virus 3C Protease Antagonizes Interferon Signaling and C142T Substitution Attenuates the FMD Virus

**DOI:** 10.3389/fmicb.2021.737031

**Published:** 2021-11-19

**Authors:** Pathum Ekanayaka, Sung Ho Shin, Prasanna Weeratunga, Hyuncheol Lee, Tae-Hwan Kim, Kiramage Chathuranga, Ashan Subasinghe, Jong-Hyeon Park, Jong-Soo Lee

**Affiliations:** ^1^College of Veterinary Medicine, Chungnam National University, Daejeon, South Korea; ^2^Animal and Plant Quarantine Agency, Gyeongsangbuk-do, South Korea; ^3^California Institute for Quantitative Biosciences, University of California, Berkeley, Berkeley, CA, United States; ^4^Infectious Disease Research Center, Korea Research Institute of Bioscience and Biotechnology, Daejeon, South Korea

**Keywords:** FMDV 3C^pro^, RIG-I, MDA5, 3C^pro^ C142T substitution, attenuated virus

## Abstract

3C protease (3C^pro^), a chymotrypsin-like cysteine protease encoded by the foot-and-mouth disease virus (FMDV), plays an essential role in processing the FMDV P1 polyprotein into individual viral capsid proteins in FMDV replication. Previously, it has been shown that 3C^pro^ is involved in the blockage of the host type-I interferon (IFN) responses by FMDV. However, the underlying mechanisms are poorly understood. Here, we demonstrated that the protease activity of 3C^pro^ contributed to the degradation of RIG-I and MDA5, key cytosolic sensors of the type-I IFN signaling cascade in proteasome, lysosome and caspase-independent manner. And also, we examined the degradation ability on RIG-I and MDA5 of wild-type FMDV 3C^pro^ and FMDV 3C^pro^ C142T mutant which is known to significantly alter the enzymatic activity of 3C^pro^. The results showed that the FMDV 3C^pro^ C142T mutant dramatically reduce the degradation of RIG-I and MDA5 due to weakened protease activity. Thus, the protease activity of FMDV 3C^pro^ governs its RIG-I and MDA5 degradation ability and subsequent negative regulation of the type-I IFN signaling. Importantly, FMD viruses harboring 3C^pro^ C142T mutant showed the moderate attenuation of FMDV in a pig model. In conclusion, our results indicate that a novel mechanism evolved by FMDV 3C^pro^ to counteract host type-I IFN responses and a rational approach to virus attenuation that could be utilized for future vaccine development.

## Introduction

Foot-and-mouth disease virus (FMDV) is a single-stranded positive-sense RNA virus (Kuhn and Wimmer, 1987; Palmenberg, 1990) belonging to genus *Aphthovirus* and family *Picornaviridae* (Belsham, 1993), and is a well-known animal viral pathogen. There are seven classified serotypes (O, A, Asia1, C, SAT1, SAT2, and SAT3) consisting of numerous subtypes (Forss et al., 1984; Grubman and Baxt, 2004) that cause contagious disease in cattle, pigs, and various cloven-hoofed animals (Sakamoto et al., 2002; Grubman and Baxt, 2004). The FMDV genome is ∼8.5 kb in size and composed of a single open reading frame (ORF) encoding a polyprotein that is proteolytically processed with the help of virus-encoded proteases into four structural proteins (VP1, VP2, VP3, and VP4) and 10 non-structural proteins (L, 2A, 2B, 2C, 3A, 3B1–3, 3C, and 3D) that accomplish distinctive functions in the viral life cycle (Forss et al., 1984; Fry et al., 2005). Among the viral-encoded proteases, 3C protease (3C^pro^) is the key enzyme, responsible for 10 of the 13 cleavages through targeting of specific sequences within the FMDV polyprotein (Skern et al., 2002; Grubman and Baxt, 2004; Belsham, 2005). Among the FMDV proteins, 3C^pro^ is the most highly conserved (82–58% identical among all serotypes) (van Rensburg et al., 2002; Birtley et al., 2005), and is the only picornaviral protease common to all genera (Curry et al., 2007).

FMDV 3C^pro^ contains two, six-stranded β-barrels coupled by a short linker. Even though the structure of the β-ribbon that forms the β-barrels of 3C^pro^ is highly conserved among all picornaviruses, it appears to be disordered in FMDV 3C^pro^ (Matthews et al., 1994; Bergmann et al., 1997; Mosimann et al., 1997; Curry et al., 2007). The β-ribbon spanning amino acid residues 138–150 of FMDV 3C^pro^ folds over the peptide binding cleft containing the active site of the enzyme. It is involved in determining the degree of flexibility of the enzyme, and influences substrate recognition through direct interaction with substrates bound in the peptide binding cleft (Dragovich et al., 1998; Matthews et al., 1999; Curry et al., 2007). Indeed, this loop functions to position the substrate correctly for proteolysis, and mutation of Cys142 at the apical tip of the β-ribbon has a momentous impact on the catalytic activity of FMDV 3C^pro^ (Curry et al., 2007; Sweeney et al., 2007).

Type-I interferons (IFNs) are major players in innate immune responses, which are considered the first line of defense against viral infection. IFN-α and IFN-β, both type-I IFNs, play a key role in triggering a robust host antiviral response to protect the host from virus infection (Durbin et al., 2000; Perry et al., 2005; Stetson and Medzhitov, 2006; Lee et al., 2019). Initially, virus evasion is detected by host cellular pattern recognition receptors (PRRs) that sense pathogen-associated molecular patterns (PAMPS). The major cytosolic PRRs, retinoic acid-inducible gene I (RIG-I), melanoma differentiation-associated gene 5 (MDA5), and toll-like receptor 3 (TLR3), all recognize viral RNA present in the cytoplasm (Uematsu and Akira, 2007; Takeuchi and Akira, 2008). MDA5 recognizes long double-stranded RNAs such as picornaviral RNA, whereas RIG-I recognizes short double-stranded RNA (dsRNA) and 5′-triphosphate single-stranded RNA with poly (U/A) motifs in RNA virus-infected cells (Yoneyama et al., 2004, 2005; Hornung et al., 2006; Pichlmair et al., 2006; Kato et al., 2008; Saito et al., 2008). Even though the sensing of picornaviral RNA is mainly mediated by MDA5, evidence also suggests a role for RIG-I (Hüsser et al., 2011). After sensing viral RNA, RIG-I and/or MDA5 interact and activate mitochondrial antiviral-signaling protein (MAVS), which leads to the activation of subsequent downstream type-I IFN signaling molecules TBK1/IKKε, IRF3, IRF7, and NF-κB (activated via IKK) to elicit antiviral responses (Akira et al., 2006; Kawai and Akira, 2006; Pichlmair and Reis e Sousa, 2007; Loo and Gale, 2011).

Foot-and-mouth disease virus is highly sensitive to type-I IFNs (Ahl, 1970; Chinsangaram et al., 1999, 2001, 2003; Summerfield et al., 2009; Dias et al., 2011), hence FMDV has evolved multiple strategies to evade host type-I IFN responses to ensure effective replication in host cells (Gao et al., 2016; Ma et al., 2018). Among FMDV proteins that interfere with the type-I IFN pathway, FMDV 3C^pro^ plays a crucial role. FMDV 3C^pro^ mediates degradation of RIG-I and MDA5 was previously identified, although this was not the main focus of the work (Wang et al., 2012; Zhu et al., 2016). However, the underlying mechanism by which FMDV 3C^pro^ mediates RIG-I and MDA5 degradation is unclear.

Based on previous studies, in the present work we revealed the exact mechanism by which FMDV 3C^pro^ mediates the degradation of RIG-I and MDA5 and demonstrated that, in the context of cellular type-I IFN signaling, the FMDV 3C^pro^ C142T substitution moderately attenuated FMDV in a pig model.

## Results

### Foot-and-Mouth Disease Virus 3C Protease Negatively Regulates Antiviral Immune Responses

Previous studies on FMDV 3C^pro^ revealed its antagonism of IFN responses (Wang et al., 2012; Du et al., 2014; Fan et al., 2018; Ma et al., 2018; Kim et al., 2021). Based on current knowledge, we confirmed the role of FMDV 3C^pro^ in the negative regulation of antiviral immune responses using wild-type FMDV 3C^pro^ of the O1/Manisa/Turkey/69 strain again.

**FIGURE 1:**
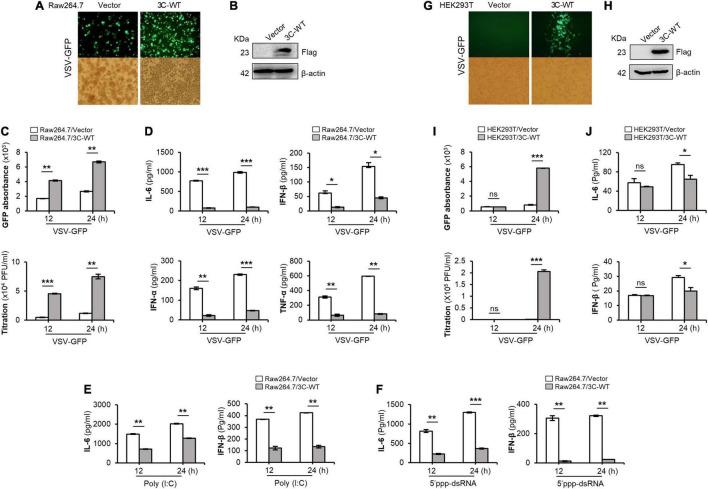
Wild-type FMDV 3C^pro^ negatively regulates type-I IFN pathway. Wild-type FMDV 3C^pro^ stably expressing or control Raw264.7 cells were infected with VSV-GFP (1 MOI) at indicated time points and, **(A)** GFP expression, **(B)** FMDV 3C^pro^ expression, **(C)** GFP absorbance and virus titer, and **(D)** IL-6, IFN-β, IFN-α, and TNF-α secretion was measured. The same cell line was treated with **(E)** poly (I:C) or **(F)** 5′PPP-dsRNA and measured their IL-6 and IFN-β secretion. HEK293T cells were transiently transfected with control plasmid or wild-type FMDV 3C^pro^ plasmid, and VSV-GFP (0.5 MOI) were infected. At indicated time points **(G)** GFP expression, **(H)** FMDV 3C^pro^ expression, **(I)** GFP absorbance and virus titer, and **(J)** IL-6, IFN-β secretion was measured at indicated time points. Data are representative of three independent experiments, each with similar results. FMDV, foot-and-mouth disease virus; IFN, interferon; IL-6, interleukin 6; TNF-α, tumor necrosis factor-alpha. All the values are expressed as mean ± SD of three biological replicates. Student’s *t*-test; *p < 0.05; **p < 0.01; ***p < 0.001; ns, not significant.

Specifically, wild-type FMDV 3C^pro^ stably expressing or control Raw264.7 cells were infected with VSV-GFP (Figures 1A,B). As expected, higher VSV-GFP expression (Figures 1A,C) and lower levels of IL-6, IFN-β, IFN-α, and TNF-α secretion (Figure 1D) were observed in Raw264.7 cells stably expressing wild-type FMDV 3C^pro^. In addition, poly (I:C) treatment (Figure 1E), 5′PPP-dsRNA treatment (Figure 1F), and PR8-GFP infection (Supplementary Figure 1) phenotypes displayed similar results in Raw264.7 cells stably expressing wild-type FMDV 3C^pro^.

Furthermore, wild-type FMDV 3C^pro^ transfected and VSV-GFP infected HEK293T cells (Figures 1G,H) showed higher virus replication (Figure 1I) and lower IL-6 and IFN-β, production (Figure 1J) than in control. In addition, PR8-GFP infection also showed similar results as VSV-GFP infection experiment in HEK293T cells upon wild-type FMDV 3C^pro^ transfection (Supplementary Figure 2). Importantly, FMDV 3C^pro^ from all seven FMDV serotypes (including Asia1 Shamir) exhibited the same phenotypes in PK15 cells (Supplementary Figure 3). These results further validate the previous findings, and suggest that FMDV 3C^pro^ is a negative regulator of type-I IFN signaling.

### Foot-and-Mouth Disease Virus 3C Protease Degrades RIG-I and MDA5 Through Its Protease Activity

**FIGURE 2:**
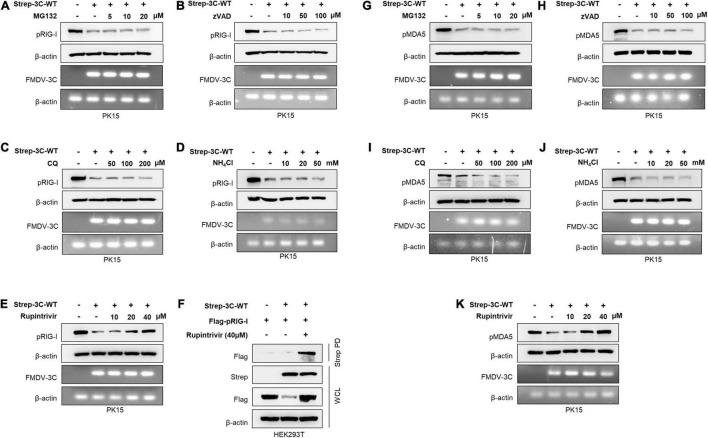
Wild-type FMDV 3C^pro^ degrades RIG-I and MDA5. PK15 cells were transiently transfected with control plasmid or wild-type FMDV 3C^pro^ in the absence or presence of **(A)** MG132 (5, 10, 20 μM), **(B)** Z-VAD (10, 50, 100 μM), **(C)** CQ (50, 100, 200 μM), **(D)** NH_4_Cl (10, 20, 50 mM), and **(E)** Rupintrivir (10, 20, 40 μM). Cell lysate was subjected to immunoblotting with antibody against RIG-I and β-actin, following the qRT-PCR for FMDV 3C^pro^ and β-actin. **(F)** HEK293T cells were cotransfected with the Flag-tagged porcine RIG-I plasmid together with control plasmid or Strep-tagged FMDV 3C^pro^ plasmid in the absence or presence of Rupintrivir. The cell lysate was subjected to Strep pulldown followed by immunoblotting with individual antibodies against Flag, Strep, or β-actin. **(G–K)** PK15 cells were transfected with control plasmid or wild-type FMDV 3C^pro^ following the EV71 infection in the absence or presence of **(G)** MG132 (5, 10, 20 μM), **(H)** Z-VAD (10, 50, 100 μM), **(I)** CQ (50, 100, 200 μM), **(J)** NH_4_Cl (10, 20, 50 mM), and **(K)** Rupintrivir (10, 20, 40 μM) as indicated above. Cell lysates were subjected to immunoblotting against individual antibodies of MDA5 and β-actin, following the qRT-PCR for FMDV 3C^pro^ and β-actin. FMDV, foot-and-mouth disease virus; EV71, enterovirus 71; RIG-I, retinoic acid-inducible gene I; MDA5, melanoma differentiation-associated protein 5; qRT-PCR, real-time quantitative reverse transcription PCR. All the data are representative of two independent experiments, each with similar results.

Based on the previous findings (Wang et al., 2012; Zhu et al., 2016), to establish the exact molecular mechanisms, FMDV 3C^pro^-mediated RIG-I and MDA5 degradation assays were conducted in the presence of different degradation pathway-related inhibitors: lysosomal inhibitors CQ and NH_4_Cl, proteasomal inhibitor MG132, pan-caspase inhibitor Z-VAD, and Rupintrivir, a broad-spectrum protease activity inhibitor of picornavirus 3C^pro^ (Wang et al., 2011; Kim et al., 2012). From the RIG-I degradation assay, the results showed that FMDV 3C^pro^ induced RIG-I degradation, but MG132 (Figure 2A), Z-VAD (Figure 2B), CQ, and NH_4_Cl (Figures 2C,D) failed to inhibit FMDV 3C^pro^-mediated RIG-I degradation. This indicates that FMDV 3C^pro^-mediated RIG-I degradation was independent of the proteasome, caspases, and lysosomes.

Next, we examined whether the protease activity of FMDV 3C^pro^ induced RIG-I degradation using the picornavirus-specific 3C^pro^ protease activity inhibitor Rupintrivir. Surprisingly, FMDV 3C^pro^-mediated RIG-I degradation was inhibited by Rupintrivir treatment in a dose-dependent manner (Figure 2E), and densitometry data further validate the results (Supplementary Figure 4). In addition, Rupintrivir inhibited FMDV 3C^pro^-mediated RIG-I degradation in the overexpression system, even with dose-dependent transfection of wild-type FMDV 3C^pro^ (Supplementary Figure 5).

Moreover, to explore the interaction between FMDV 3C^pro^ and RIG-I, immunoprecipitation assays were conducted by separately transfecting HEK293T cells with a Flag-tagged porcine RIG-I plasmid together with a control plasmid or Strep-tagged wild-type FMDV 3C^pro^ plasmid, and then treating them with and without Rupintrivir. Interestingly, the co-immunoprecipitation results showed a clear association between FMDV 3C^pro^ and RIG-I (Figure 2F).

Since MDA5 is the main cellular sensor for FMDV detection in the type-I IFN pathway, similar to RIG-I, MDA5 degradation assays were conducted in PK15 cells. Similar to previous findings (Kim et al., 2021), MG132 (Figure 2G), Z-VAD (Figure 2H), CQ, and NH_4_Cl (Figures 2I,J) did not inhibit MDA5 degradation, suggesting that FMDV 3C^pro^-mediated MDA5 degradation is independent of proteasome, caspase, and lysosome pathways. However, as expected, a dose-dependent treatment of Rupintrivir completely inhibited FMDV 3C^pro^-mediated MDA5 degradation (Figure 2K). These results suggest that FMDV 3C^pro^-mediated RIG-I and MDA5 degradation is governed by the protease activity of FMDV 3C^pro^.

**FIGURE 3:**
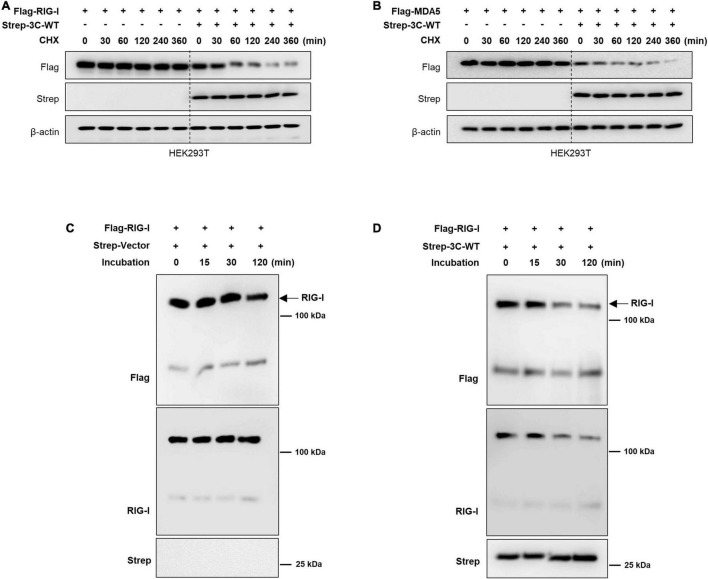
Wild-type FMDV 3C^pro^ mediated RIG-I and MDA5 degradation is related to its protease activity. HEK293T cells were transiently transfected with **(A)** Flag-tagged RIG-I or **(B)** MDA5 plasmid together with Strep-tagged control plasmid or Strep-tagged wild-type FMDV 3C^pro^. At 24 h after transfection of plasmids, cells were treated with Cycloheximide (CHX) and cells were harvested at indicated time points after CHX treatment. Cell lysates were subjected to immunoblotting with individual antibodies against Flag, Strep, and β-actin. For the wild-type FMDV 3C^pro^ mediated *in vitro* RIG-I degradation, RIG-I protein (2 μg) was incubated with **(C)** control or **(D)** with FMDV-3C wild-type protein (2 μg) in the *in vitro* degradation buffer containing 50 mM HEPES-KOH pH 7.5, 35 mM KCl and 1 mM DTT. Reactions were carried out at 37°C for 0–2 h and terminated by adding an equal volume of SDS-PAGE sample buffer and heating at 100°C for 5 min. The samples were subjected to immunoblotting with anti-Flag, -RIG-I, and -Strep antibodies.

Importantly, the FMDV 3C^pro^ is known for inhibiting the host protein synthesis by cleaving the eIF4AI and eIF4G translation initiation factors (Belsham et al., 2000; Li et al., 2001). Hence, here we did the cycloheximide (CHX) chase assay to investigate whether FMDV 3C^pro^ degrade RIG-I and MDA5 through direct cleavage or by host protein synthesis inhibition. Based on the results, even after the treatment of CHX the RIG-I and MDA5 protein levels tend to degrade when the presence of wild-type FMDV 3C^pro^ protein (Figures 3A,B). Since CHX is a protein synthesis inhibitor (Siegel and Sisler, 1963; Ennis and Lubin, 1964), the results in Figures 3A,B explain that the degradation of RIG-I and MDA5 expression level are not directly related to the wild-type FMDV 3C^pro^-mediate cleavage of eIF4AI and eIF4G translation initiation factors. This is further confirmed by the *in vitro* degradation assay results (Figures 3C,D) which do not involve any translation process. Hence, we assumed that wild-type FMDV 3C^pro^ degrade RIG-I and MDA5 through direct cleavage. However, in the degradation assay results (Figure 2 and Supplementary Figure 5) which have used C-terminal Flag-tagged RIG-I and MDA5 plasmids, and C-terminal region detecting RIG-I antibody, we could not observe any cleavage band of RIG-I or MDA5. Because of that, we suggest that FMDV 3C^pro^ involve in the cleavage of RIG-I and MDA5 at multiple locations which prevents the detection of cleavage bands. Hence to further validate our suggestion, wild-type FMDV 3C^pro^-mediated RIG-I and MDA5 degradation assay was conducted with the N-terminal GFP-tagged RIG-I and MDA5 plasmids. Based on the results, GFP-fused RIG-I and MDA5 tend to cleavage and degradation by allowing to detection of cleaved GFP protein upon dose-dependent transfection of wild-type FMDV 3C^pro^ (Supplementary Figure 6). Collectively, these results suggest that the wild-type FMDV 3C^pro^ directly cleave RIG-I and MDA5 at multiple locations including both N- and C-terminal regions which caused their reduced expression level (degradation), and this degradation is not directly related to the FMDV 3C^pro^-mediate cleavage of eIF4AI and eIF4G translation initiation factors.

### Foot-and-Mouth Disease Virus 3C Protease C142T Mutation Blocks Interferon Antagonist Ability

The Cys142 residue of FMDV 3C^pro^ located at the apical tip of the β-ribbon is known to play a crucial role in catalytic activity and substrate recognition of the enzyme (Curry et al., 2007; Sweeney et al., 2007). Importantly, C142T substitution was shown to result in a significant reduction in protease activity of FMDV 3C^pro^ (Sweeney et al., 2007). Since the protease activity of FMDV 3C^pro^ governs RIG-I and MDA5 degradation, in the present study we constructed the FMDV 3C^pro^ C142T point mutant, which we expected to lack IFN antagonist ability.

**FIGURE 4:**
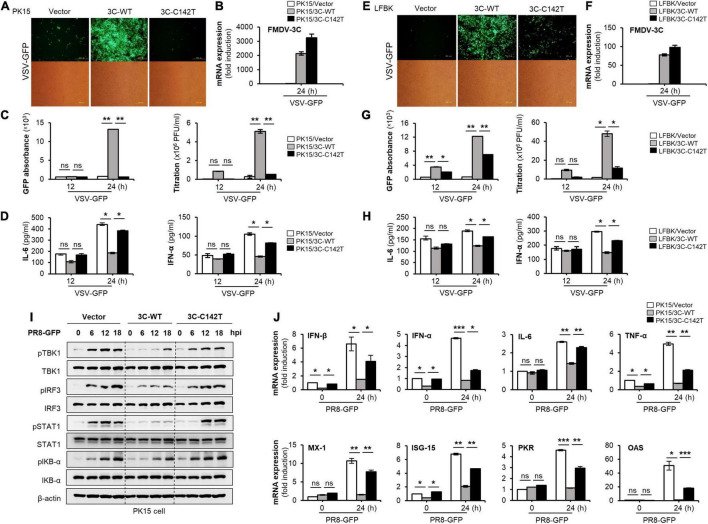
FMDV 3C^pro^ C142T is deficient in the negative regulation of type-I IFN signaling. The PK15 cells or LFBK cells were transiently transfected with wild-type FMDV 3C^pro^, 3C^pro^ C142T, or control plasmid, following VSV-GFP (1 MOI) infection. **(A,E)** GFP expression, **(B,F)** wild-type FMDV 3C^pro^ and 3C^pro^ C142T expression by qRT-PCR, **(C,G)** GFP absorbance and virus titer, **(D,H)** IL-6 and IFN-α secretion was measured at indicated time points. **(I)** PK15 cells were transiently transfected with wild-type FMDV 3C^pro^, 3C^pro^ C142T, or control plasmid and infected with PR8-GFP (3 MOI). Immunoblotting was performed with cells harvested at 0, 6, 12, and 18 hpi to detect phosphorylated (p-) TBK1, TBK1, p-IRF3, IRF3, p-STAT1, STAT1, p-IκB-α, IkB-α, and β-actin. **(J)** PK15 cells were transiently transfected with wild-type FMDV 3C^pro^, 3C^pro^ C142T, or control plasmid and PR8-GFP (3 MOI) infection, following total RNA extraction and qRT-PCR for respective antiviral genes as mentioned. Data are representative of three independent experiments each with similar results. FMDV, foot-and-mouth disease virus; IFN, interferon; IL-6, interleukin 6; TBK1, TANK binding kinase 1; IRF3, interferon regulatory factor 3; STAT1, signal transducer and activator of transcription 1; IκB, inhibitor of nuclear factor-kappa B. All the values are expressed as mean ± SD of at least two biological replicates. Student’s *t*-test; *p < 0.05; **p < 0.01; ***p < 0.001; ns, not significant.

For comparison of antiviral immune evasion ability, PK15 cells and LFBK cells were transiently transfected with control plasmid or plasmids containing wild-type FMDV 3C^pro^ or FMDV 3C^pro^ C142T, and then were infected with VSV-GFP. As expected, in PK15 cells, VSV-GFP expression was lower, and IL-6 and IFN-α production was higher in cells expressing FMDV 3C^pro^ C142T than in cells expressing wild-type FMDV 3C^pro^, relative to control (Figures 4A–D). This similar phenomenon was observed in LFBK cells (Figures 4E–H), wild-type FMDV 3C^pro^ or FMDV 3C^pro^ C142T stably expressing Raw264.7 cells, and BHK21 cells (Supplementary Figure 7).

To further examine the effect of FMDV 3C^pro^ on virus-mediated activation of the type-I IFN signal cascade, we examined virus-induced phosphorylation of TBK1, IRF3, STAT1, and IKB-α, all of which are key signaling molecules in type-I IFN and NF-κB pathways. Specifically, control, wild-type FMDV 3C^pro^, and FMDV 3C^pro^ C142T plasmids were separately transfected into PK15 cells infected with PR8-GFP, and cells were harvested at 0, 6, 12, and 18 hpi for immunoblot analysis. Interestingly, phosphorylation of TBK1, IRF3, STAT1, and IKB-α was lower in PK15 cells expressing wild-type FMDV 3C^pro^ than in PK15 cells expressing FMDV 3C^pro^ C142T or control cells (Figure 4I). In addition, wild-type FMDV 3C^pro^ inhibited the expression of mRNAs of IFN-β, IFN-α, IL-6, and other antiviral-related genes (Figure 4J). However, FMDV 3C^pro^ C142T did not affect the phosphorylation of type-I IFN or NF-κB pathway-associated molecules, or the mRNA expression levels of antiviral genes (Figures 4I,J). Moreover, phosphorylation of type-I IFN or NF-κB pathway-related molecules and mRNA expression levels of antiviral genes were lower in Raw264.7 cells stably expressing wild-type FMDV 3C^pro^ than in FMDV 3C^pro^ C142T stably expressing Raw264.7 or control cells (Supplementary Figure 8). These results suggest that wild-type FMDV 3C^pro^ is a negative regulator of virus-induced type-I IFN signaling, and the FMDV 3C^pro^ C142T mutation blocks this antagonistic ability.

### The Foot-and-Mouth Disease Virus 3C Protease C142T Mutant Does Not Degrade RIG-I and MDA5

Since the FMDV 3C^pro^ C142T is known for its significant reduction of protease activity (Sweeney et al., 2007), and the protease activity of FMDV 3C^pro^ governs the RIG-I and MDA5 degradation, the effect of FMDV 3C^pro^ C142T on RIG-I and MDA5 degradation was compared with respect to wild-type FMDV 3C^pro^.

**FIGURE 5:**
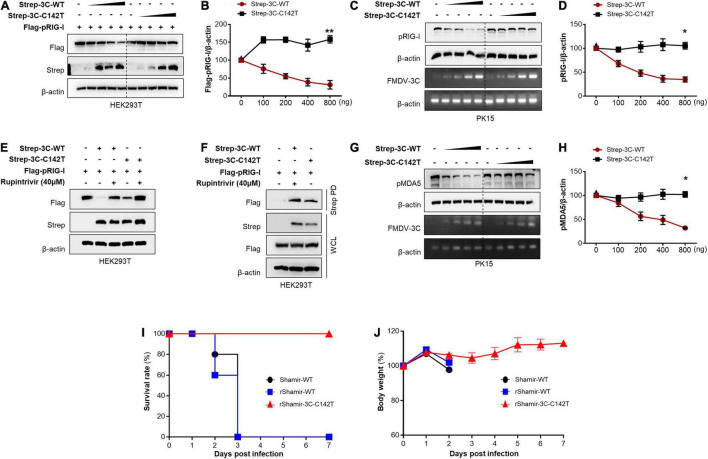
FMDV 3C^pro^ C142T is not involved in RIG-I and MDA5 degradation. **(A)** HEK293T cells were transiently transfected with Strep-tagged wild-type FMDV 3C^pro^ or 3C^pro^ C142T plasmids in a dose-dependent manner, or control plasmid, together with Flag-tagged porcine RIG-I plasmid. Cell lysates were subjected to immunoblotting with individual antibodies against Flag, Strep, and β-actin, and **(B)** band intensity was measured. **(C)** PK15 cells were transiently transfected with Strep-tagged wild-type FMDV 3C^pro^ or 3C^pro^ C142T plasmids in a dose-dependent manner, or control plasmid. Cell lysates were subjected to immunoblotting with individual antibody against RIG-I, or β-actin, following the qRT-PCR for FMDV 3C^pro^, 3C^pro^ C142T, and β-actin, and **(D)** band intensity was measured. **(E)** HEK293T cells were transfected with a control plasmid, or Strep-tagged wild-type FMDV 3C^pro^, or 3C^pro^ C142T plasmid, together with a Flag-tagged porcine RIG-I plasmid in the absence or presence of Rupintrivir. **(F)** HEK293T cells were transiently transfected with Flag-tagged porcine RIG-I plasmid, control plasmid, or Strep-tagged wild-type FMDV 3C^pro^ or FMDV 3C^pro^ C142T plasmid, and subsequent treatment of Rupintrivir into the selected combination. Cell lysates were subjected to Strep pull-down and immunoblotted with individual antibodies against Flag, Strep, and β-actin. **(G)** PK15 cells were transiently transfected with Strep-tagged wild-type FMDV 3C^pro^ or 3C^pro^ C142T plasmids in a dose-dependent manner, or control plasmid. And EV71 virus was infected. Cell lysates were subjected to immunoblotting with individual antibodies against MDA5 or β-actin, following the qRT-PCR for FMDV 3C^pro^, 3C^pro^ C142T, and β-actin, and **(H)** band intensity was measured. For the comparison of pathogenesis in adult mice, 7 weeks old C57 female mice were divided into three groups (*n* = 5) and intraperitoneally infected with Shamir-WT, rShamir-WT or rShamir-3C-C142T at a concentration of 5 × 10^4^.^0^ TCID_50_/0.1 mL, and **(I)** survival rates and **(J)** body weight changes were measured. FMDV, foot-and-mouth disease virus; EV71, enterovirus 71; RIG-I, retinoic acid-inducible gene I; MDA5, melanoma differentiation-associated protein 5; qRT-PCR, real-time quantitative reverse transcription PCR; WT, wild-type. All the western blot data are representative of two independent experiments and the values are expressed as mean ± SD of at least two biological replicates. Student’s *t*-test; *p < 0.05; **p < 0.01.

To examine the impact of FMDV 3C^pro^ C142T on RIG-I degradation, HEK293T cells were transfected with Strep-tagged wild-type FMDV 3C^pro^ or FMDV 3C^pro^ C142T plasmids in a dose-dependent manner, or control plasmid, together with Flag-tagged porcine RIG-I plasmid. Cell lysates were then immunoblotted separately with antibodies against Flag, Strep, or β-actin (Figures 5A,B). In addition, endogenous RIG-I degradation assays were performed in PK15 cells by transient transfection of wild-type FMDV 3C^pro^ or FMDV 3C^pro^ C142T plasmids in a dose-dependent manner, or control plasmid (Figures 5C,D). The results showed that RIG-I was vulnerable to degradation by wild-type FMDV 3C^pro^, but FMDV 3C^pro^ C142T did not degrade RIG-I because its protease activity was abrogated.

To further investigate whether the inability of FMDV 3C^pro^ C142T to degrade RIG-I was directly correlated with its abridged protease activity, HEK293T cells were transfected with control, Strep-tagged wild-type FMDV 3C^pro^, or FMDV 3C^pro^ C142T plasmids, together with a Flag-tagged porcine RIG-I plasmid. Thereafter, cells were treated with Rupintrivir in selected combinations, and cell lysates were subjected to immunoblotting with antibodies against Flag, Strep, or β-actin. The results revealed strong degradation of RIG-I upon overexpression of wild-type FMDV 3C^pro^, and Rupintrivir treatment alleviated this effect. Moreover, RIG-I degradation upon FMDV 3C^pro^ C142T transfection was significantly lower than that following wild-type FMDV 3C^pro^ transfection, and Rupintrivir treatment further stabilized RIG-I levels by alleviating the remaining protease activity of 3C^pro^ C142T. This suggests that the abridged protease activity of FMDV 3C^pro^ C142T (Sweeney et al., 2007) was directly responsible for its inability to degrade RIG-I (Figure 5E).

In addition, an immunoprecipitation assay was conducted to explore the interaction between wild-type FMDV 3C^pro^ and FMDV 3C^pro^ C142T with RIG-I. The results showed that irrespective of the point mutation and the resulting abrogation of protease activity, FMDV 3C^pro^ C142T interacted with RIG-I similarly to wild-type FMDV 3C^pro^ (Figure 5F).

Moreover, the influence of FMDV 3C^pro^ C142T on MDA5 degradation was analyzed with respect to wild-type FMDV 3C^pro^. Specifically, PK15 cells were transiently transfected with wild-type FMDV 3C^pro^ or FMDV 3C^pro^ C142T plasmids in a dose-dependent manner, or with control plasmid, and cells were infected with EV71 at 24 h post-transfection to induce MDA5 production (Loo et al., 2008). After 18 hpi, cell lysates were immunoblotted separately with antibodies against MDA5 or β-actin, followed by qRT-PCR analysis of FMDV 3C^pro^, FMDV 3C^pro^ C142T, and β-actin. The immunoblot results revealed that, unlike wild-type FMDV 3C^pro^, FMDV 3C^pro^ C142T was not involved in MDA5 degradation (Figures 5G,H). Collectively, these results explain why FMDV 3C^pro^ C142T lacks IFN antagonist ability.

Based on these findings, we extended our study to examine the effect of 3C^pro^ C142T on FMDV pathogenicity. Therefore, other than the wild-type FMD virus (Shamir-WT), two recombinant FMDVs were constructed harboring wild-type 3C^pro^ (rShamir-WT) or 3C^pro^ C142T (rShamir-3C-C142T), and *in vivo* evaluation of virulence was performed in adult mice. Mice were separately infected with Shamir-WT, rShamir-WT, and rShamir-3C-C142T, and survival rates and body weight changes were monitored. Interestingly, consistent with *in vitro* virus replication results, mice infected with rShamir-3C-C142T survived until 7 dpi, and all mice infected with Shamir-WT or rShamir-WT died at 3 dpi (Figure 5I). Furthermore, in the same mice used for survival tests, body weight was measured continuously up to 7 dpi, but compared with mice infected with Shamir-WT and rShamir-WT, significant weight loss was not observed in mice infected with rShamir-3C-C142T (Figure 5J).

### Foot-and-Mouth Disease Virus Harboring 3C Protease C142T Display Moderately Attenuated Virulence in Pigs

**FIGURE 6:**
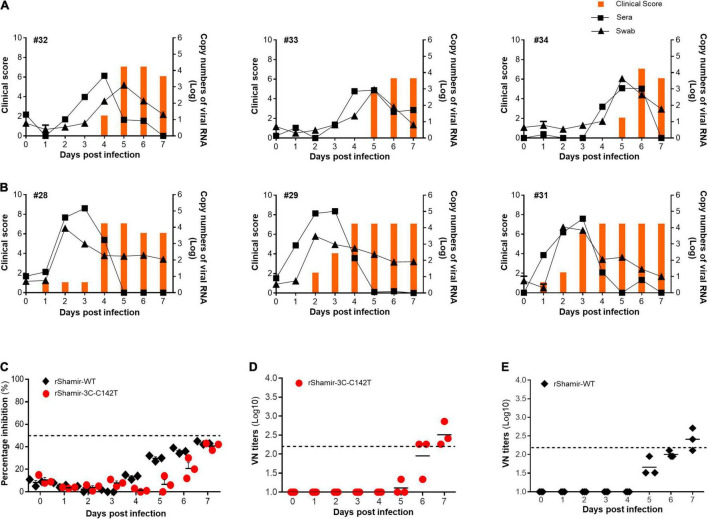
Pathogenesis of the recombinant viruses in pigs. The experiment was carried out with six pigs which were divided into two groups (*n* = 3). **(A)** One group was infected with rShamir-3C-C142T (animals #32, #33, and #34) and the **(B)** other group was infected with rShamir-WT (animals #28, #29, and #31), intradermally at a concentration of 1 × 10^3^.^0^ TCID_50_/0.1 mL. The right Y-axis of the graph shows the amount of virus in sera and swab as log10 values and the left Y-axis shows the clinical index. **(C)** percentage inhibition and **(D,E)** virus-neutralizing titer of each recombinant FMDV infected animals in two groups were measured.

Finally, the pathogenicity of rShamir-3C-C142T was evaluated in its natural host. Specifically, pigs were directly infected with rShamir-WT or rShamir-3C-C142T. Following infection, several disease parameters (clinical score, viremia, percentage inhibition, and neutralizing antibody titers) were analyzed. Based on the results, both rShamir-WT and rShamir-3C-C142T showed clinical signs typical of FMD. However, animals (#32, #33, and #34) infected with rShamir-3C-C142T took longer to show clinical signs (starting at 5 dpi on average), and a considerable level of virus release (visible at 4 dpi) was evident (Figure 6A), while animals infected with rShamir-WT (#28, #29, and #31) showed clinical signs within 1 dpi and a considerable level of virus release within 2 dpi (Figure 6B). In addition, SP antibody levels of both groups were increased comparably at 5 and 6 dpi (Figure 6C). Furthermore, neutralizing antibody titers of rShamir-3C-C142T and rShamir-WT groups were similarly increased at 6 and 5 dpi, respectively (Figures 6D,E). These results confirmed the moderately attenuated nature of FMDV in the swine model upon 3C^pro^ C142T substitution.

Together, these results demonstrate that the C142T substitution in 3C^pro^ abrogates its ability to degrade RIG-I and MDA5 by its protease activity and consequently showed the attenuation of rShamir-3C-C142T in adult mice and moderate attenuation in pigs.

## Discussion

Type-I IFN antiviral responses are capable of controlling most if not all virus infections, and picornaviruses are no exception (Delneste et al., 2007; Lee et al., 2019). In response, FMDV has evolved multiple antagonistic strategies to evade cellular type-I IFN responses and facilitate rapid infection of host cells (Ma et al., 2018).

The 3C^pro^ enzyme, the main protease in FMDV, plays a crucial role in immune evasion by targeting key molecules in antiviral signal transduction pathways. For example, FMDV 3C^pro^ cleaves NEMO, resulting in suppression of the NF-κB pathway (Wang et al., 2012), and negative regulation of both autophagy and NF-κB antiviral responses through degradation of ATG5-ATG12 cellular proteins (Fan et al., 2018). In addition, STAT1 nuclear translocation is disrupted via degradation of KPNA1 nuclear translocation signal receptor by FMDV 3C^pro^ (Du et al., 2014). Furthermore, FMDV 3C^pro^-mediated lysosomal degradation of host antiviral effector PKR and subsequent facilitation of virus replication have been reported (Li et al., 2017). Importantly, the degradation of RIG-1, MDA5 (Wang et al., 2012; Zhu et al., 2016; Kim et al., 2021), and LGP2 (Zhu et al., 2017) by FMDV 3C^pro^ directly affects FMDV sensing by the type-I IFN pathway. However, the underlying molecular mechanisms remain unknown. In addition, MDA5 degradation and RIG-I cleavage during different Picornavirus infection was reported previously, but they haven’t investigated about the FMDV (Barral et al., 2007, 2009).

In the present study, we revealed the exact mechanism of FMDV 3C^pro^-mediated degradation of RIG-I and MDA5 in the type-I IFN pathway, and examined the impact of 3C^pro^ C142T substitution on FMDV pathogenicity. First, we showed that overexpression of wild-type FMDV 3C^pro^ in epithelial and macrophage cells reduced RNA virus-induced IFN-β and proinflammatory cytokine production, resulting in increased virus replication, as shown previously (Wang et al., 2012; Du et al., 2014; Zhu et al., 2016; Fan et al., 2018; Kim et al., 2021). Second, using Rupintrivir, a broad-spectrum picornavirus 3C protease activity inhibitor (Wang et al., 2011; Kim et al., 2012), and with other experiments demonstrated that the protease activity of FMDV 3C^pro^ governs the degradation of RIG-I and MDA5 by direct cleavage. Third, we constructed the FMDV 3C^pro^ C142T point mutant, which is much less active than the wild-type enzyme (Sweeney et al., 2007), and assessed the impact of the mutation on virus replication and RIG-I and MDA5 degradation. The C142T mutation did not have a positive impact on induction of virus replication compared with the wild-type enzyme, further confirming that 3C^pro^ C142T lacked the ability to degrade RIG-I and MDA5. Fourth, we generated recombinant FMD virus harboring the 3C^pro^ C142T mutant (rShamir-3C-C142T) and compared its pathogenicity alongside that of FMDV harboring wild-type 3C^pro^ (rShamir-WT) in mice and pigs. The results revealed moderate attenuation of FMDV in a pig model upon C142T substitution.

After confirming the IFN inhibitory phenotypes upon wild-type FMDV 3C^pro^ expression, we explored the exact mechanism behind 3C^pro^-mediated RIG-I and MDA5 degradation. Previous studies reported degradation of RIG-I and MDA5 by FMDV 3C^pro^ (Wang et al., 2012; Zhu et al., 2016; Kim et al., 2021), but the exact mechanism is unclear. Most 3C^pro^-mediated immune evasion mechanisms are related to its protease activity (Wang et al., 2012; Du et al., 2014), except for PKR degradation (Li et al., 2017), which proceeds via the lysosomal pathway. We therefore hypothesized that 3C^pro^-mediated RIG-I and MDA5 degradation might result from the protease activity of 3C^pro^. To test this, we used Rupintrivir, a broad-spectrum picornavirus 3C protease activity inhibitor (Kim et al., 2012), and the results confirmed our hypothesis (Figure 2). Rupintrivir inhibits the protease activity of Enterovirus 71 (EV71) 3C^pro^ (Wang et al., 2011) and also inhibits the protease activity of other picornaviral 3C^pro^ enzymes, including that of FMDV, with varying IC_50_ values (Robinson et al., 2016). Rupintrivir can neutralize the negative charge of the Glu71 residue of EV71 3C^pro^ following its interaction with Arg39. This neutralization subsequently assists the deprotonation of His40 of 3C^pro^ during proteolysis, which blocks the protease activity of 3C^pro^ (Wang et al., 2011).

Upon FMDV infection, the RIG-I and MDA5 detect the FMDV RNA (Loo et al., 2008; Hüsser et al., 2011) and transmit the activation signal to produce type-I IFNs through the type-I IFN signal cascade (McNab et al., 2015). During the signal transmission in the type-I IFN signaling cascade, type-I IFN pathway-associated molecules undergo posttranslational modifications and among those modifications, phosphorylation plays a major role (Doly et al., 1998; Chiang and Gack, 2017). Since FMDV is very sensitive to the type-I IFNs (Chinsangaram et al., 2003; Dias et al., 2011), FMDV 3C^pro^ degrades the RIG-I and MDA5 as demonstrated in our study for the effective replication of FMDV in the host. This leads to the inhibition of type-I IFN cascade and subsequent type-I IFN production, and it is revealed by the phosphorylation reduction of TBK1, IRF3, and STAT1. Since the type-I IFN pathway links to the NF-κB pathway by the TRAF family member proteins (Furr and Marriott, 2012), IKB-α also showed a phosphorylation reduction when the presence of FMDV 3C^pro^ (Figure 4).

In the previous paper, FMDV 3C^pro^ is known for cleaving the eIF4AI and eIF4G translation initiation factors to shut off the host protein synthesis (Belsham et al., 2000; Li et al., 2001). Hence, this also could lead to the reduction of RIG-I and MDA5 protein expression when the presence of FMDV 3C^pro^. However, the results in Figure 3 and Supplementary Figure 6 explain that the degradation of RIG-I and MDA5 expression level is not directly related to the FMDV 3C^pro^-mediate cleavage of eIF4AI and eIF4G translation initiation factors. Instead of that, we suggest that FMDV 3C^pro^ involve in the cleavage of RIG-I and MDA5 at multiple locations including both N- and C-terminal regions which result in their degradation. Also, we assume that the multiple cleavages of RIG-I and MDA5 are the reason for not detecting cleavage band of RIG-I or MDA5 in our degradation experiments. In addition, if there is a highly significant impact of FMDV 3C^pro^ mediated eIF4AI and eIF4G cleavage on RIG-I and MDA5 expression levels due to the translation inhibition, there should be a reduction in the expression levels of type-I IFN pathway associated other proteins. But, based on our results, we could not observe such reduction of type-I IFN pathway associated other protein (TBK1, IRF3, and STAT1) expression levels (Figure 4I and Supplementary Figure 8) when the presence of FMDV 3C^pro^.

FMDV 3C^pro^ is a trypsin-like serine protease that possesses a Cys-His-Asp catalytic triad in its active site (Birtley and Curry, 2005; Yin et al., 2005; Sweeney et al., 2007). The loop formed by residues 138 − 150 of FMDV 3C^pro^ folds into a β-ribbon structure that is positioned on top of the N-terminal region of the substrate binding cleft (Dragovich et al., 1998; Matthews et al., 1999; Curry et al., 2007). The 13 residues forming the β-ribbon in FMDV 3C^pro^ is remarkably similar to that of the 3C^pro^ enzyme of human rhinovirus (HRV) (Matthews et al., 1994) and poliovirus (PV) (Mosimann et al., 1997). However, the β-ribbon of FMDV 3C^pro^ is one amino acid longer than the β-ribbons of HRV and PV 3C^pro^, and this additional residue is located at the apical tip of the β-ribbon. The tips of the β-ribbons have higher structural variation, as evidenced by higher B factors, indicating that the β-ribbon is a highly flexible feature on the surface of FMDV 3C^pro^ (Sweeney et al., 2007). Hence, residue 142 located at the tip of the β-ribbon in FMDV 3C^pro^ strongly influences its enzymatic activity and substrate recognition (Curry et al., 2007; Sweeney et al., 2007; Zunszain et al., 2010). Residue 142 of FMDV 3C^pro^ maps closely to L127 in HRV2 3C^pro^, suggesting that the relatively apolar C142 in wild-type FMDV 3C^pro^ functions similarly to the hydrophobic L127 in HRV2 3C^pro^ (Sweeney et al., 2007). The C142L mutant of FMDV 3C^pro^ is known to exhibit a higher level of enzymatic activity than the wild-type enzyme. Therefore, the apolar residue at position 142 in FMDV 3C^pro^ is important for optimal activity (Sweeney et al., 2007). Moreover, the interaction of FMDV 3C^pro^ with the P4 and P2 residues of the substrate is governed by the apolarity of the amino acid in position 142, since interactions with this residue determine presentation of the substrate to the active site of the enzyme in the correct orientation for proteolysis (Sweeney et al., 2007).

In addition, amino acid substitutions at position 142 in FMDV 3C^pro^ affect its solubility (Sweeney et al., 2007). The insertion of a polar side chain via the C142S substitution almost completely inhibits FMDV 3C^pro^ protease activity and can lead to total inactivation of the virus. Similarly, introducing a less polar residue at position 142 (Ala, Val, or Thr) results in different levels of enzymatic activity (Sweeney et al., 2007). Since our final purpose was to develop a live-attenuated FMD virus, C142S is not suitable due to total inactivation of the virus. Thus, the C142T substitution, the second-best substitution to C142S for abrogating protease activity, which reduces the enzymatic activity by more than 60% compared with wild-type FMDV 3C^pro^, was used for this purpose.

FMDV 3C^pro^-mediated IFN suppression is known to result from various molecular mechanisms, most of which are related to its protease activity (Wang et al., 2012; Du et al., 2014). Since FMDV 3C^pro^ C142T has significantly less protease activity (Sweeney et al., 2007), it has the potential to abolish the immune suppression mechanisms related to protease activity, including RIG-I and MDA5 degradation. Indeed, FMDV 3C^pro^ C142T did not exhibit type-I IFN suppression-related phenotypes (Figure 4). The RIG-I and 3C^pro^ C142T immunoprecipitation results revealed that amino acid substitution at position 142 did not affect the ability to bind substrate (Figure 5F). However, the C142T substitution affected the ability of the β-ribbon to present substrates in the correct orientation for proteolysis (Sweeney et al., 2007). Therefore, even though there is no difference in substrate binding, FMDV 3C^pro^ C142T lacks RIG-I and MDA5 degradation activity because it cannot present substrates in the correct orientation for proteolysis (Figure 5).

Consequently, rShamir-3C-C142T displayed a significant decrease in pathogenicity in mice (Figures 5I,J). Similarly, previous studies showed that mutations in viral proteins cause defects in their IFN suppressive functions, resulting in attenuated viral pathogenicity. For example, E96A/E97A mutations in influenza A virus NS1 fail to suppress TRIM25-mediated IFN responses, leading to avirulence in mice (Gack et al., 2009). Moreover, Ebola virus VP35 protein with K319A/R322A point mutations causes attenuation in guinea pigs due to its inability to suppress IFN responses (Prins et al., 2010). Additionally, A30P substitution in the NS2A non-structural protein of West Nile virus causes attenuation in mice because it fails to inhibit type-I IFN induction (Liu et al., 2006). Furthermore, we previously showed that FMDV VP1 E83K substitution results in both defective IFN suppression and receptor alteration, leading to virus attenuation (Ekanayaka et al., 2020). Based on this evidence, we suggest that the resultant avirulence of rShamir-3C-C142T in mice (Figures 5I,J) is due to its inability to suppress type-I IFN responses.

Importantly, rShamir-3C-C142T showed a moderate level of attenuation in the pig model. rShamir-3C-C142T took longer to cause clinical signs and significant viremia than rShamir-WT (Figures 6A,B). Since pigs are the natural hosts of FMDV, IFN suppressive viral proteins of FMDV can more effectively target IFN-related molecules for suppression in pigs than in mice.

In summary, our results showed that FMDV 3C^pro^ is an IFN antagonist that degrades RIG-I and MDA5 through its protease activity. This adds another layer of complexity to the antagonistic strategies implemented by an economically important viral pathogen to evade the immune responses of its hosts. Furthermore, the 3C^pro^ C142T point mutant was defective for IFN suppressive functions, resulting in moderate attenuation of the virus in a pig model. These findings may assist the generation of a highly successful live-attenuated virus by incorporating IFN suppression-deficient mutations into other immune-suppressive FMDV proteins together with 3C^pro^ C142T, which could provide a new basis for the development of future FMDV vaccines.

## Materials and Methods

### Cell Culture

HEK293T (ATCC CRL11268), mouse leukemic monocyte macrophage (RAW264.7; ATCC TIB-71), PK-15 (ATCC CCL-33), LF-BK (RRID:CVCL_RX26), BHK-21 (ATCC CCL-10), and Vero (ATCC CCL-81) cells were cultured in Dulbecco’s modified Eagle’s medium (DMEM-high glucose; Gibco, California, United States) containing 10% heat-inactivated fetal bovine serum (FBS; Gibco) and 1% antibiotic/antimycotic solution (Gibco). Cells were incubated at 37°C under a 5% CO_2_ atmosphere.

### Antibodies and Inhibitors

For immunoblot analysis, each individual antibody against Strep (2-1509-001) was purchased from IBA Life Sciences. Antibody against GFP (sc-9996) and β-actin (sc-47778) was from Santa Cruz Biotechnology. Antibodies for Flag (M2; 8146), RIG-I (D14G6; 3743), MDA-5 (D74E4; 5321), phospho-TBK1/NAK (D52C2; 5483), TBK-1 (D1B4; 3504), phospho-IRF3 (4D4G; 4947), IRF3 (D83B9; 4302), phospho-STAT1 (58D6; 9167), STAT1 (42H3; 9175), phospho-IκBα (14D4; 2859), and IκBα (9242) were purchased from Cell Signaling Technology.

The inhibitors MG132 (M8699), chloroquine (C6628), ammonium chloride (A9434), and Rupintrivir (PZ0315) were purchased from Sigma-Aldrich, and Z-VAD-FMK (sc-3067) was from Santa Cruz Biotechnology.

### Plasmid Construction

Porcine RIG-I was amplified by standard reverse transcription (RT)-PCR using the cDNA obtained from total RNA extracted from PK15 cells and cloned into the pIRES-Flag expression vector (Wang et al., 2008, 2010). To construct full-length wild-type FMDV 3C^pro^ and the 3C^pro^ C142T point mutant of the O1/Manisa/Turkey/69 strain, gene-specific PCR primers were used, and the product was cloned into IRES-Flag and pEXPR-STrEP expression vectors. The IFN-β promoter and luciferase reporter plasmids were generated as described previously (Kim et al., 2017).

### Virus Infection and Plasmid Transfection

GFP-expressing H1N1 influenza A virus (A/PR8/8/34; PR8-GFP) was propagated in allantoic fluid from 10-day-old embryonated chicken eggs, and GFP-expressing vesicular stomatitis virus (VSV-GFP) was propagated in Vero cells. Propagated viruses were titrated by plaque assay. Before virus infection of cells, the culture medium was changed to DMEM containing 1% FBS and 1% antibiotic-antimycotic, and target cells were infected based on multiplicity of infection (MOI). After a 2 h incubation at 37°C, extracellular virus was removed and replaced with 10% FBS containing DMEM. Plasmids were transfected into HEK293T, RAW264.7, PK15, LFBK, and BHK-21 cells using Lipofectamine 2000 (Invitrogen) according to the manufacturer’s protocol.

### Virus Titer Determination

GFP-expressing virus-infected cell culture supernatants and cells were collected at the indicated times, and virus titers were measured by plaque assay using *Ceropithecus aethiops* epithelial kidney (Vero) cells. A monolayer of Vero cells was seeded in 12-well plates, incubated for 12 h, and cells were inoculated for 2 h with serially diluted virus-containing culture supernatants with 1% DMEM. After a 2 h incubation, solutions were removed and replaced with DMEM containing 0.1% agarose (Sigma). Plates were then incubated at 37°C for another 36 h and examined for plaque formation under 200 × magnification. Virus titer was calculated using the number of plaque-forming units and the dilution factor.

### Enzyme-Linked Immunosorbent Assay

Enzyme-linked immunosorbent assay (ELISA) was performed to detect secreted inflammatory cytokines in cell culture supernatants. Human IL-6 (BD Biosciences, 555220), human interferon-β (CUSABIO, CSB-E09889h), mouse IL-6 (BD Biosciences, 555240), mouse interferon-β (CUSABIO, CSB-E04945m), mouse interferon-α (PBL Assay Science, 42120-1), mouse TNF-α (BD Biosciences, 555268), porcine IL-6 (R&D Systems, P6000B), and porcine IFN-α (CUSABIO, CSB-E07328p) were used for analysis according to the manufacturer’s protocols.

### Quantitative Real-Time PCR

Total RNA was isolated from cells using an RNeasy Mini Kit (Qiagen), and cDNA was synthesized using reverse transcriptase (Toyobo). The qRT-PCR analysis was performed using a QuantiTect SYBR Green PCR Kit (Toyobo) according to the manufacturer’s instructions with the primers listed in Table 1, on a Rotorgene instrument (Qiagen). The mRNA expression levels were analyzed according to the delta-delta CT (2^–ΔΔCT^) method, and glyceraldehyde-3-phosphate dehydrogenase (GAPDH) or β-actin were used as internal housekeeping genes for normalization.

**TABLE 1:** Primers for qRT-PCR.

**Gene^a^**	**Forward**	**Reverse**
pIFN-β	AAATCGCTCTCCTGATGTGT	TGCTCCTTTGTTGGTATCG
pIFN-α	GCCTCCTGCACCAGTTCTACA	TGCATGACACAGGCTTCCA
pIL-6	CACCGGTCTTGTGGAGTTTC	GTGGTGGCTTTGTCTGGATT
pTNF-α	CCACGTTGTAGCCAATGTC	CTGGGAGTAGATGAGGTACAG
pMX-1	TAGGCAATCAGCCATACG	GTTGATGGTCTCCTGCTTAC
pISG-15	AAATCGCTCTCCTGATGTGT	TGCTCCTTTGTTGGTATCG
pPKR	GAGAAGGTAGAGCGTGAAG	CCAGCAACCGTAGTAGAG
pOAS	CTGTCGTTGGACGATGTATGCT	CAGCCGGGTCCAGAATCA
pβ-actin	CTCGATCATGAAGTGCGACG	GTGATCTCCTTCTGCATCCTGT
mIFN-β	TCCAAGAAAGGACGAACATTCG	TGCGGACATCTCCCACGTCAA
mIL-6	TCCATCCAGTTGCCTTCTTGG	CCACGATTTCCCAGAGAACATG
mTNF-α	AGCAAACCACCAAGTGGAGGA	GCTGGCACCACTAGTTGGTTGT
mISG-15	CAATGGCCTGGGACCTAAA	CTTCTTCAGTTCTGACACCGTCAT
mISG-20	AGAGATCACGGACTACAGAA	TCTGTGGACGTGTCATAGAT
mISG-56	AGAGAACAGCTACCACCTTT	TGGACCTGCTCTGAGATTCT
mOAS	GAGGCGGTTGGCTGAAGAGG	GAGGAAGGCTGGCTGTGATTGG
mADAR1	CCAAAGACACTTCCTCTC	CAGTGTGGTGGTTGTACT
mGAPDH	TGACCACAGTCCATGCCATC	GACGGACACATTGGGGGTAG
FMDV-3C	TCTTCGCGGAGAAGTACGAC AAGAT	CTGAGAGCATGTCCTGTCCTT TTAC

*^a^p, porcine; m, mouse.*

### Strep Pull-Down and Immunoprecipitation Assay

At 36 h post-transfection of target plasmids, cells were harvested and lysed by radio-immunoprecipitation assay (RIPA) lysis buffer (50 mM Tris-HCl, 150 mM NaCl, 0.5% sodium deoxycholate, 1% IGEPAL, 1 mM NaF, 1 mM Na_3_VO_4_) containing protease inhibitor cocktail and phosphatase inhibitor cocktail (Sigma) to generate whole cell lysates (WCLs). WCLs were incubated with Sepharose 6B resin (GE Healthcare Life Science) at 4°C for 2 h. Following this pre-clearing step, for Strep pull-down, WCLs were incubated for 12 h with a 50% slurry of Strep-Tactin Sepharose Strep beads (IBA Solutions for Life Sciences, Germany). Immunoprecipitated beads were collected by centrifugation and washed with lysis buffer for immunoblot analysis.

### Immunoblot Analysis

Cells were washed with phosphate-buffered saline (PBS) and lysed in RIPA lysis buffer in the presence of protease inhibitor cocktail and phosphatase inhibitor cocktail (Sigma). Samples were separated by SDS-PAGE and transferred onto a PVDF membrane (Bio-Rad) using a Trans-Blot semi-dry transfer cell (Bio-Rad) with buffer containing 30 mM Tris, 200 mM glycine, and 20% methanol. Membranes were blocked for 1 h in Tris-buffered saline containing 0.05% Tween 20 (TBST) and 5% bovine serum albumin (BSA), and proteins were probed with the target antibody in 5% BSA-TBST. Following overnight incubation at 4°C, membranes were washed three times with PBS containing 0.05% Tween (PBST) or TBST for 10 min each. Following this washing step, membranes were treated with horseradish peroxidase (HRP)-conjugated secondary antibody for 1 h at room temperature. The washing step was repeated three more times (10 min each), and HRP was visualized using an Enhanced Chemiluminescence Detection System (GE Life Sciences) and an LAS-4000 Mini Lumino Image Analyzer (GE Life Sciences).

### RIG-I and MDA5 Degradation Assay in PK15 Cells

RIG-I and MDA5 degradation assays were conducted in the presence of different degradation pathway-related inhibitors: lysosomal inhibitors CQ and NH_4_Cl, proteasomal inhibitor MG132, pan-caspase inhibitor Z-VAD, and Rupintrivir, a broad-spectrum protease activity inhibitor of picornavirus 3C^pro^.

For the RIG-I degradation assay, PK15 cells were transiently transfected with control plasmid or wild-type FMDV 3C^pro^ plasmid and treated with the inhibitors in a dose-dependent manner. Cell lysates were subjected to immunoblotting with each individual antibody against RIG-I or β-actin, followed by qRT-PCR analysis of FMDV 3C^pro^ and β-actin.

For MDA5 degradation assay, PK15 cells were transiently transfected with control plasmid or Strep-tagged wild-type FMDV 3C^pro^ plasmid. At 24 h post-transfection, cells were infected with Enterovirus 71 (EV71) to induce MDA5 production. Inhibitors were then applied at 12 h post-infection (hpi), and 6 h later cells were harvested for immunoblotting using antibodies against MDA5 or β-actin, followed by qRT-PCR for FMDV 3C^pro^ and β-actin.

### Cycloheximide Chase Assay

HEK293T cells were transfected with either Flag-tagged RIG-I or MDA5 expressing plasmid together with the Strep-tagged control plasmid or Strep-tagged FMDV-3C wild-type plasmid. At the 24 h post-transfection, CHX (50 μg/mL) were treated according to the previous publications (Li et al., 2015; Yoo et al., 2015) to inhibit further protein synthesis, and cells were harvested at indicated time points after CHX treatment. The harvested cells were lysed and subjected to immunoblotting with respective antibodies.

### *In vitro* Degradation Assay

First, RIG-I and FMDV-3C protein samples were prepared by immunoprecipitation. For that, HEK293T cells were separately transfected with Flag-tagged RIG-I, Strep-tagged FMDV-3C wild-type, or Strep-tagged control (empty vector) plasmid. The cells were harvested at 36 h post-transfection and whole-cell lysates (WCL) were subjected for the immunoprecipitation, and fused target proteins were recovered by elution with 100 mM Glycine Buffer Solution, pH 2–2.5 (Santa Cruz Biotechnology, sc-295018), and neutralized by 500 mM NH_4_HCO_3_. Second, *in vitro* degradation assay was conducted according to the previous publications with some modifications (Lin et al., 2012; Eaglesham et al., 2019). For that, eluted proteins were quantified, and RIG-I protein (2 μg) was incubated with FMDV-3C wild-type protein (2 μg) or with the eluted samples of the Strep-tagged control plasmid transfected cells (control), in the *in vitro* degradation buffer containing 50 mM HEPES-KOH pH 7.5, 35 mM KCl and 1 mM DTT. Reactions were carried out at 37°C for 0–2 h and terminated by adding an equal volume of SDS-PAGE sample buffer and heating at 100°C for 5 min. The samples were subjected to immunoblotting with respective antibodies.

### Virus Rescue

The complete genome of the Asia1 Shamir serotype of FMDV was inserted into the cloning vector to produce the rShamir-WT recombinant virus. The rShamir-3C-C142T recombinant virus was produced by mutating cysteine (C) 142 to threonine (T) in the rShamir-WT 3Cpro using a KOD-Plus-Mutagenesis Kit (Toyobo). The cloned plasmids were linearized by treatment with the restriction enzyme *Spe*I (NEB). These linearized plasmids were transfected into baby hamster kidney (BHK) T7-9 cells stably expressing T7 RNA polymerase using Lipofectamine 2000 (Invitrogen) to recover recombinant viruses. These viruses were then amplified in the ZZ-R fetal goat tongue cell line for isolation of recombinant viruses (Brehm et al., 2009).

### Pathogenesis in Mice

Seven weeks old C57 female mice were divided into three groups (*n* = 5) and infected with Shamir-WT, rShamir-WT or rShamir-3C-C142T, intraperitoneally at a concentration of 5 × 10^4^.^0^ TCID_50_/0.1 mL. After the infection, survival rates and weight changes in C57 mice were observed until the 7 days post infection.

### Pathogenesis in Pigs

Six 90-day-old Yucatan pigs were randomly divided into two groups and separately challenged with rShamir-WT or rShamir-3C-C142T. Each virus was infected by intradermal injection at a concentration of 1 × 10^3^.^0^ TCID_50_/0.1 mL. Following the challenge, viremia was analyzed from sera and swabs of infected animals from 0 days post-challenge (dpc) to 10 dpc, and clinical symptoms were also monitored. For viremia analysis, viral RNAs were extracted from sera and swab samples, and real-time RT-PCR was performed using specific primers. The clinical score was determined as follows: an elevated body temperature of 40°C (1 point), >40.5°C (2 points), or >41°C (3 points); reduced appetite (1 point) or no food intake and food leftover from the day before (2 points); lameness (1 point) or reluctance to stand (2 points); presence of heat and pain after palpation of the coronary band (1 point) or not standing on the affected foot (2 points); vesicles on feet, dependent on the number of feet affected, with a maximum of 4 points; and visible mouth lesions on the tongue (1 point), gums, or lips (1 point), or snout (1 point), with a maximum of 3 points (Alves et al., 2009).

### Enzyme-Linked Immunosorbent Assay for the Detection of Foot-and-Mouth Disease Virus Structural Protein Antibodies (Percent Inhibition)

Antibodies to the structural proteins of FMDV in sera were detected using PrioCheck FMDV Asia1 (Prionics, Switzerland) according to the manufactures instructions. When the samples reflected percent inhibition values of ≥50%, the animals were regarded as having demonstrated an immune response.

### Virus Neutralization Test

Serum samples were collected from pigs after infection and heat-inactivated at 56°C for 30 min. Next, test serum was incubated with FMDV at 100 TCID_50_ for 1 h, and LFBK cells were added to the plate and incubated for 3 days. The CPE was checked to determine the titers, which were calculated as log_10_ of the reciprocal antibody dilution required to neutralize 100 TCID_50_ of the virus.

### Graphing and Statistical Analysis

Graph plotting and all statistical analyses were performed using GraphPad Prism software version 6 for Windows. Data are presented as means ± standard deviation (SD) of two biological replicates, and are representative of at least three independent experiments. Unpaired *t*-tests were performed at each time point to compare the control and treatment groups (^∗^*p* < 0.05; ^∗∗^*p* < 0.01; ^∗∗∗^*p* < 0.001).

## Data Availability Statement

The original contributions presented in the study are included in the article/Supplementary Material, further inquiries can be directed to the corresponding authors.

## Ethics Statement

All the animal experiments including the pigs experiment were carried in strict accordance with the recommendations of the Guide for the Care and Use of Laboratory Animals of the Animal and Plant Quarantine Agency (APQA), Republic of Korea (approval no. 2019-462). All the FMDV related animal experiments were carried out under biosecurity and safety precautions. All efforts were made to minimize animal suffering.

## Author Contributions

PE and SS performed most of the experiments. PW, HL, T-HK, KC, and AS helped with the experiments and contributed to the discussions. PE, J-HP, and J-SL designed the study. PE and J-SL wrote the manuscript. J-HP and J-SL supervised the study. All authors helped with the data analysis.

## Conflict of Interest

The authors declare that the research was conducted in the absence of any commercial or financial relationships that could be construed as a potential conflict of interest.

## Publisher’s Note

All claims expressed in this article are solely those of the authors and do not necessarily represent those of their affiliated organizations, or those of the publisher, the editors and the reviewers. Any product that may be evaluated in this article, or claim that may be made by its manufacturer, is not guaranteed or endorsed by the publisher.
