# Supplementary material for: Foot-and-Mouth Disease Virus 3C Protease Antagonizes Interferon Signaling and C142T Substitution Attenuates the FMD Virus
Source: Front Microbiol. 2021 Nov 19;12:737031. doi: 10.3389/fmicb.2021.737031 (PMC8639872; doi:10.3389/fmicb.2021.737031)
Supplement: Supplementary file 1 [file Presentation_1.PPTX]

## Slide 1
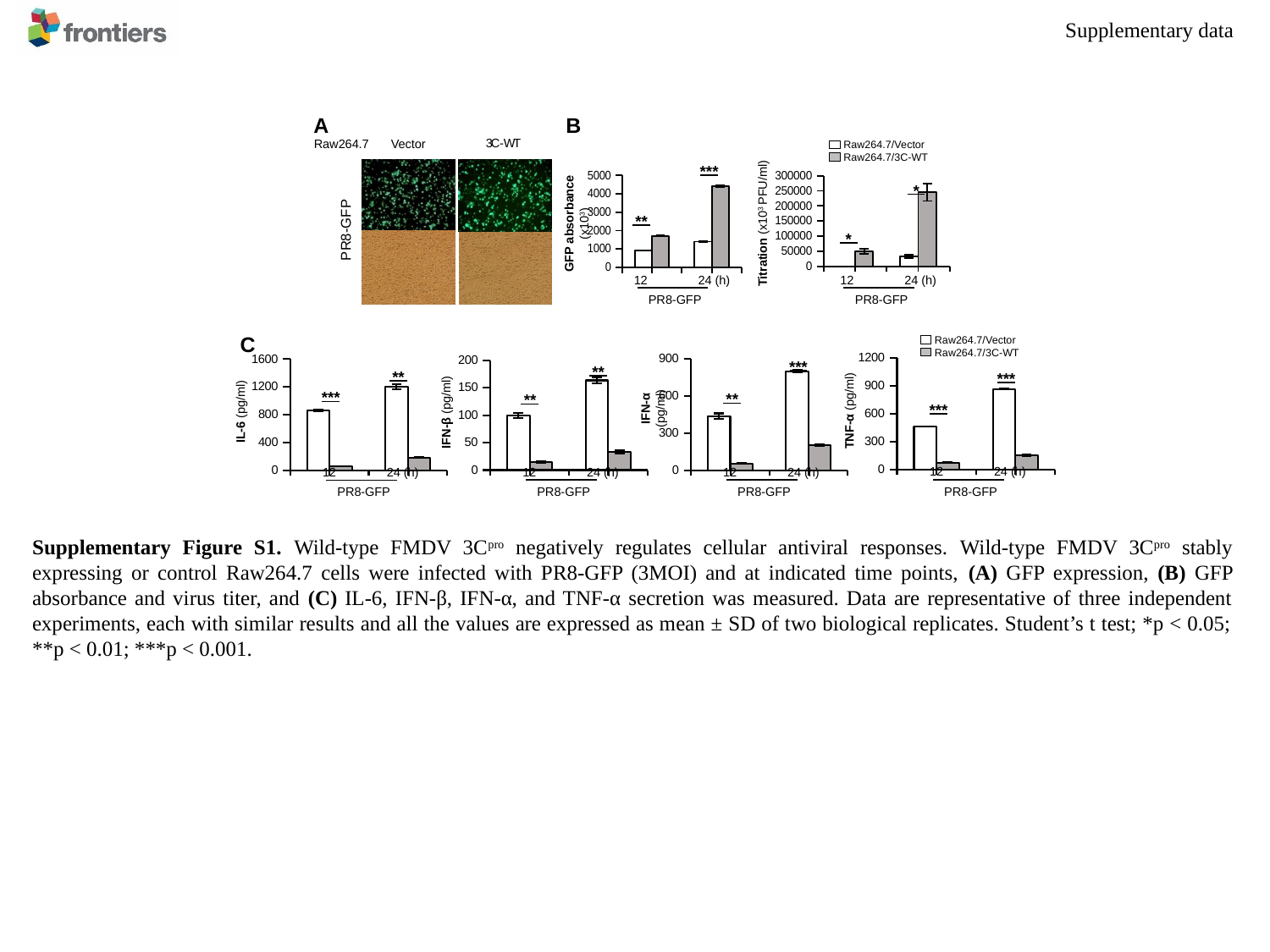

Supplementary data
B
A
Raw264.7
Vector
3C - WT
PR8-GFP
Raw264.7/Vector
Raw264.7/3C-WT
Titration (x103 PFU/ml)
### Chart
| Category | IRES | 3C-21 |
|---|---|---|
| 12 hpi | 0.0 | 49500.0 |
| 24 hpi | 32500.0 | 245000.0 | 12 24 (h)
PR8-GFP
*
*
GFP absorbance (x103)
### Chart
| Category | IRES | 3C -21 |
|---|---|---|
| 12 hpi | 933.4856666666685 | 1714.58 |
| 24 hpi | 1403.6299999999999 | 4436.9800000000005 | 12 24 (h)
PR8-GFP
***
**
C
Raw264.7/Vector
Raw264.7/3C-WT
### Chart
| Category | | |
|---|---|---|
| 12h | 464.005 | 73.09 |
| 24h | 865.64 | 152.05 |TNF-α (pg/ml)
 12 24 (h)
PR8-GFP
***
***
### Chart
| Category | | |
|---|---|---|
| 12h | 435.73 | 53.29 |
| 24h | 797.1949999999999 | 202.185 |IFN-α (pg/ml)
 12 24 (h)
PR8-GFP
***
**
### Chart
| Category | | |
|---|---|---|
| 12h | 861.675 | 57.215 |
| 24h | 1199.2 | 180.405 |IL-6 (pg/ml)
 12 24 (h)
PR8-GFP
**
***
### Chart
| Category | | |
|---|---|---|
| 12h | 99.14500000000001 | 14.19 |
| 24h | 163.06 | 32.95 |IFN-β (pg/ml)
 12 24 (h)
PR8-GFP
**
**
Supplementary Figure S1. Wild-type FMDV 3Cpro negatively regulates cellular antiviral responses. Wild-type FMDV 3Cpro stably expressing or control Raw264.7 cells were infected with PR8-GFP (3MOI) and at indicated time points, (A) GFP expression, (B) GFP absorbance and virus titer, and (C) IL-6, IFN-β, IFN-α, and TNF-α secretion was measured. Data are representative of three independent experiments, each with similar results and all the values are expressed as mean ± SD of two biological replicates. Student’s t test; *p < 0.05; **p < 0.01; ***p < 0.001.

## Slide 2
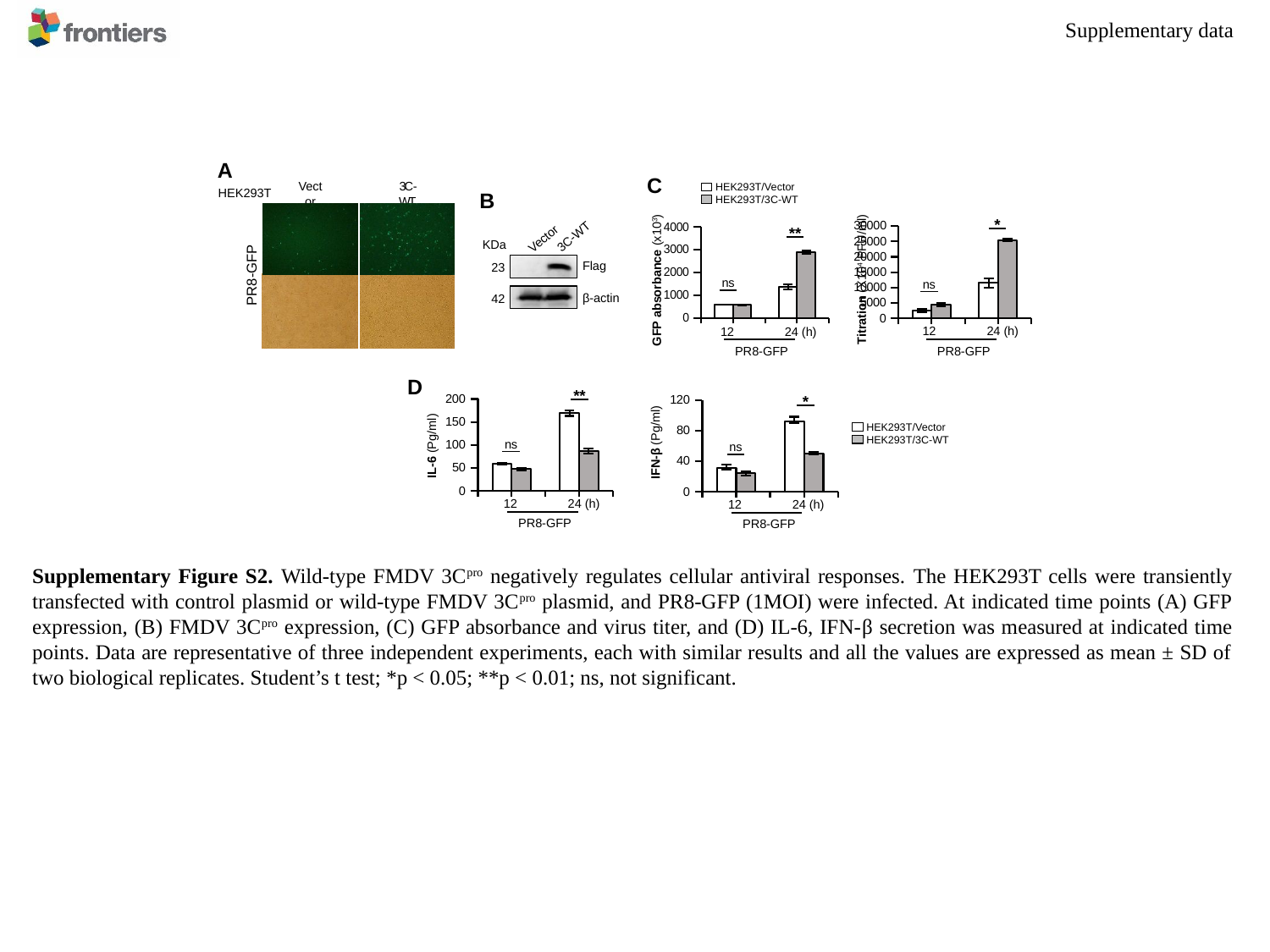

Supplementary data
A
C
3C - WT
Vector
PR8-GFP
HEK293T
HEK293T/Vector
HEK293T/3C-WT
B
**
### Chart
| Category | | |
|---|---|---|
| 12h | 579.53 | 582.875 |
| 24h | 1367.69 | 2900.15 |GFP absorbance (x103)
ns
 12 24 (h)
PR8-GFP
*
### Chart
| Category | | |
|---|---|---|
| 12h | 2500.0 | 4500.0 |
| 24h | 11500.0 | 25500.0 |Titration (X104 PFU/ml)
ns
 12 24 (h)
PR8-GFP
3C-WT
Vector
Flag
23
β-actin
42
KDa
D
**
### Chart
| Category | | |
|---|---|---|
| 12h | 58.705 | 48.150000000000006 |
| 24h | 169.015 | 86.975 |ns
IL-6 (Pg/ml)
 12 24 (h)
PR8-GFP
*
### Chart
| Category | | |
|---|---|---|
| 12h | 31.67 | 24.255000000000003 |
| 24h | 92.595 | 50.705 |IFN-β (Pg/ml)
ns
 12 24 (h)
PR8-GFP
HEK293T/Vector
HEK293T/3C-WT
Supplementary Figure S2. Wild-type FMDV 3Cpro negatively regulates cellular antiviral responses. The HEK293T cells were transiently transfected with control plasmid or wild-type FMDV 3Cpro plasmid, and PR8-GFP (1MOI) were infected. At indicated time points (A) GFP expression, (B) FMDV 3Cpro expression, (C) GFP absorbance and virus titer, and (D) IL-6, IFN-β secretion was measured at indicated time points. Data are representative of three independent experiments, each with similar results and all the values are expressed as mean ± SD of two biological replicates. Student’s t test; *p < 0.05; **p < 0.01; ns, not significant.

## Slide 3
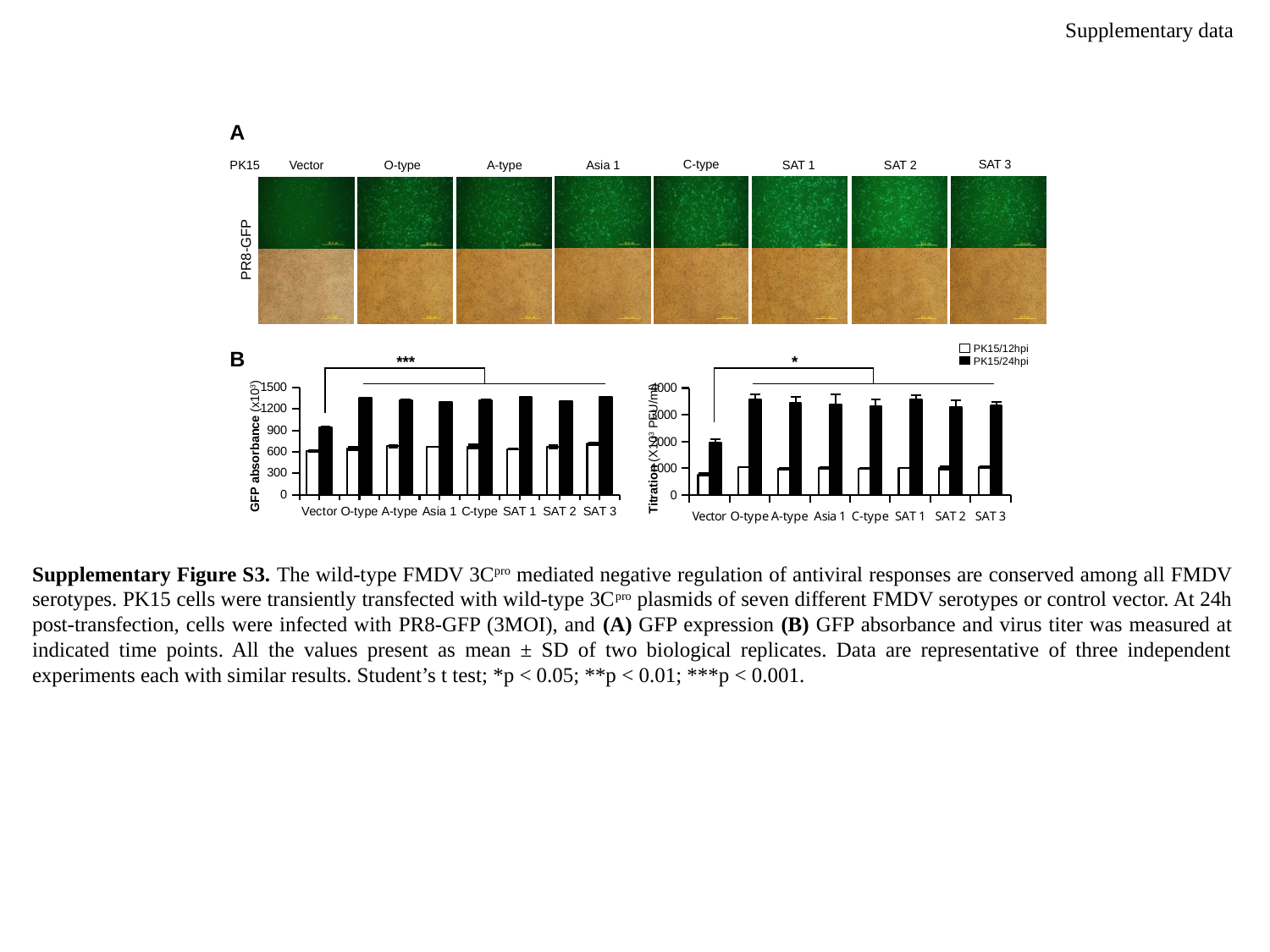

Supplementary data
A
C-type
SAT 3
Vector
PK15
O-type
A-type
Asia 1
SAT 2
SAT 1
PR8-GFP
PK15/12hpi
PK15/24hpi
B
***
### Chart
| Category | 12hpi | 24hpi |
|---|---|---|
| Vector | 611.7165 | 946.675 |
| O-type | 647.24 | 1356.6999999999998 |
| A-type | 676.8100000000001 | 1315.49 |
| Asia 1 | 666.76 | 1297.97 |
| C-type | 675.85 | 1325.9650000000001 |
| SAT 1 | 639.6 | 1364.665 |
| SAT 2 | 670.965 | 1307.565 |
| SAT 3 | 711.505 | 1363.6799999999998 |GFP absorbance (x103)
*
### Chart
| Category | 12hpi | 24hpi |
|---|---|---|
| Vector | 767.5 | 1960.0 |
| O-type | 1040.0 | 3565.0 |
| A-type | 975.0 | 3430.0 |
| Asia 1 | 1017.0 | 3370.0 |
| C-type | 983.0 | 3305.0 |
| SAT 1 | 1009.5 | 3565.0 |
| SAT 2 | 1006.0 | 3275.0 |
| SAT 3 | 1031.5 | 3335.0 |Titration (X103 PFU/ml)
Supplementary Figure S3. The wild-type FMDV 3Cpro mediated negative regulation of antiviral responses are conserved among all FMDV serotypes. PK15 cells were transiently transfected with wild-type 3Cpro plasmids of seven different FMDV serotypes or control vector. At 24h post-transfection, cells were infected with PR8-GFP (3MOI), and (A) GFP expression (B) GFP absorbance and virus titer was measured at indicated time points. All the values present as mean ± SD of two biological replicates. Data are representative of three independent experiments each with similar results. Student’s t test; *p < 0.05; **p < 0.01; ***p < 0.001.

## Slide 4
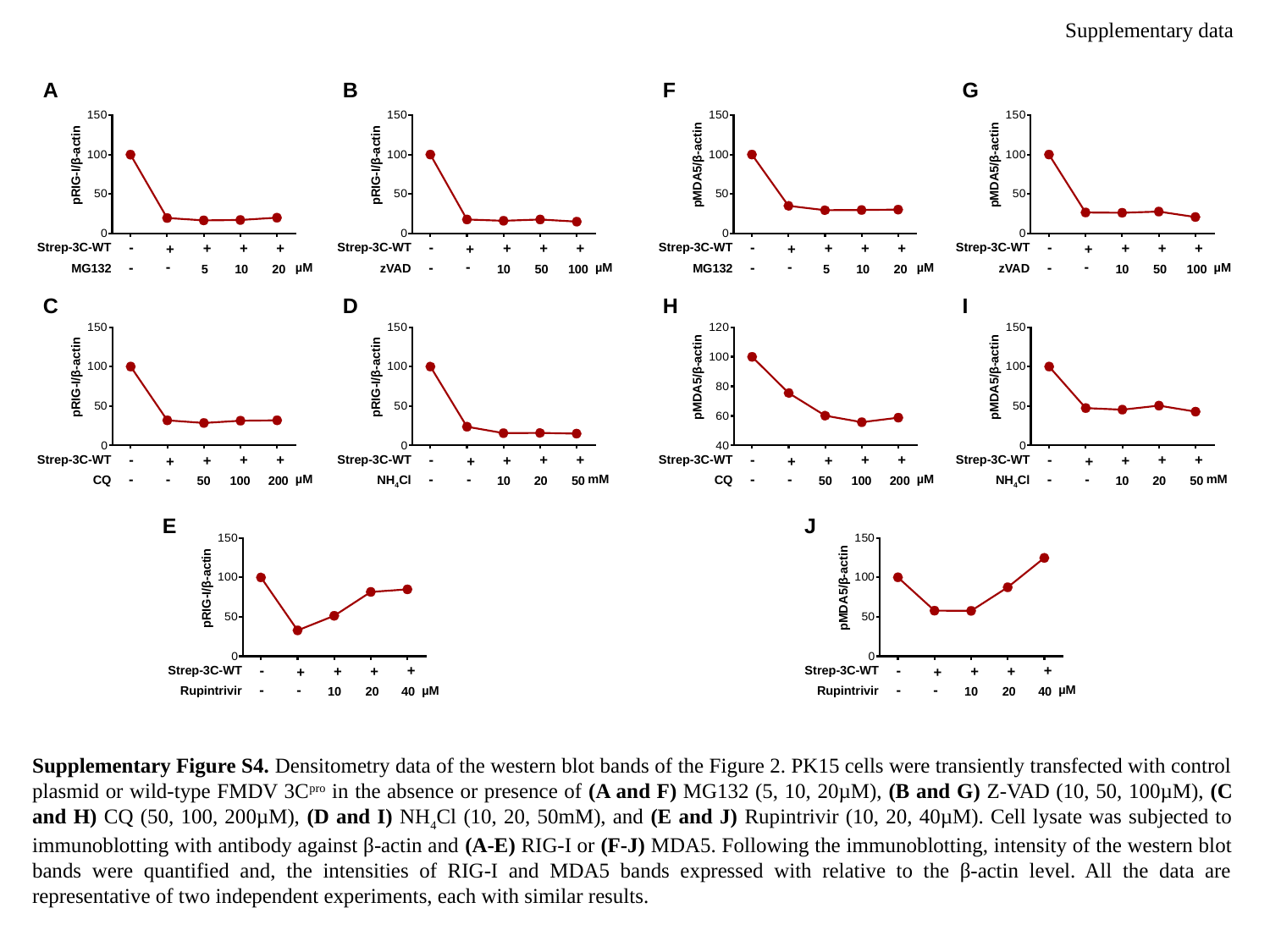

Supplementary data
A
B
pRIG-I/β-actin
-
+
+
Strep-3C-WT
+
+
-
-
µM
MG132
5
10
20
pRIG-I/β-actin
-
+
+
Strep-3C-WT
+
+
-
-
µM
zVAD
10
50
100
C
D
pRIG-I/β-actin
-
+
+
Strep-3C-WT
+
+
-
-
mM
NH4Cl
10
20
50
pRIG-I/β-actin
-
+
+
Strep-3C-WT
+
+
-
-
µM
CQ
50
100
200
E
pRIG-I/β-actin
-
+
+
Strep-3C-WT
+
+
-
-
µM
Rupintrivir
10
20
40
F
G
pMDA5/β-actin
-
+
+
Strep-3C-WT
+
+
-
-
µM
MG132
5
10
20
pMDA5/β-actin
-
+
+
Strep-3C-WT
+
+
-
-
µM
zVAD
10
50
100
H
I
pMDA5/β-actin
-
+
+
Strep-3C-WT
+
+
-
-
µM
CQ
50
100
200
pMDA5/β-actin
-
+
+
Strep-3C-WT
+
+
-
-
mM
NH4Cl
10
20
50
J
pMDA5/β-actin
-
+
+
Strep-3C-WT
+
+
-
-
µM
Rupintrivir
10
20
40
Supplementary Figure S4. Densitometry data of the western blot bands of the Figure 2. PK15 cells were transiently transfected with control plasmid or wild-type FMDV 3Cpro in the absence or presence of (A and F) MG132 (5, 10, 20µM), (B and G) Z-VAD (10, 50, 100µM), (C and H) CQ (50, 100, 200µM), (D and I) NH4Cl (10, 20, 50mM), and (E and J) Rupintrivir (10, 20, 40µM). Cell lysate was subjected to immunoblotting with antibody against β-actin and (A-E) RIG-I or (F-J) MDA5. Following the immunoblotting, intensity of the western blot bands were quantified and, the intensities of RIG-I and MDA5 bands expressed with relative to the β-actin level. All the data are representative of two independent experiments, each with similar results.

## Slide 5
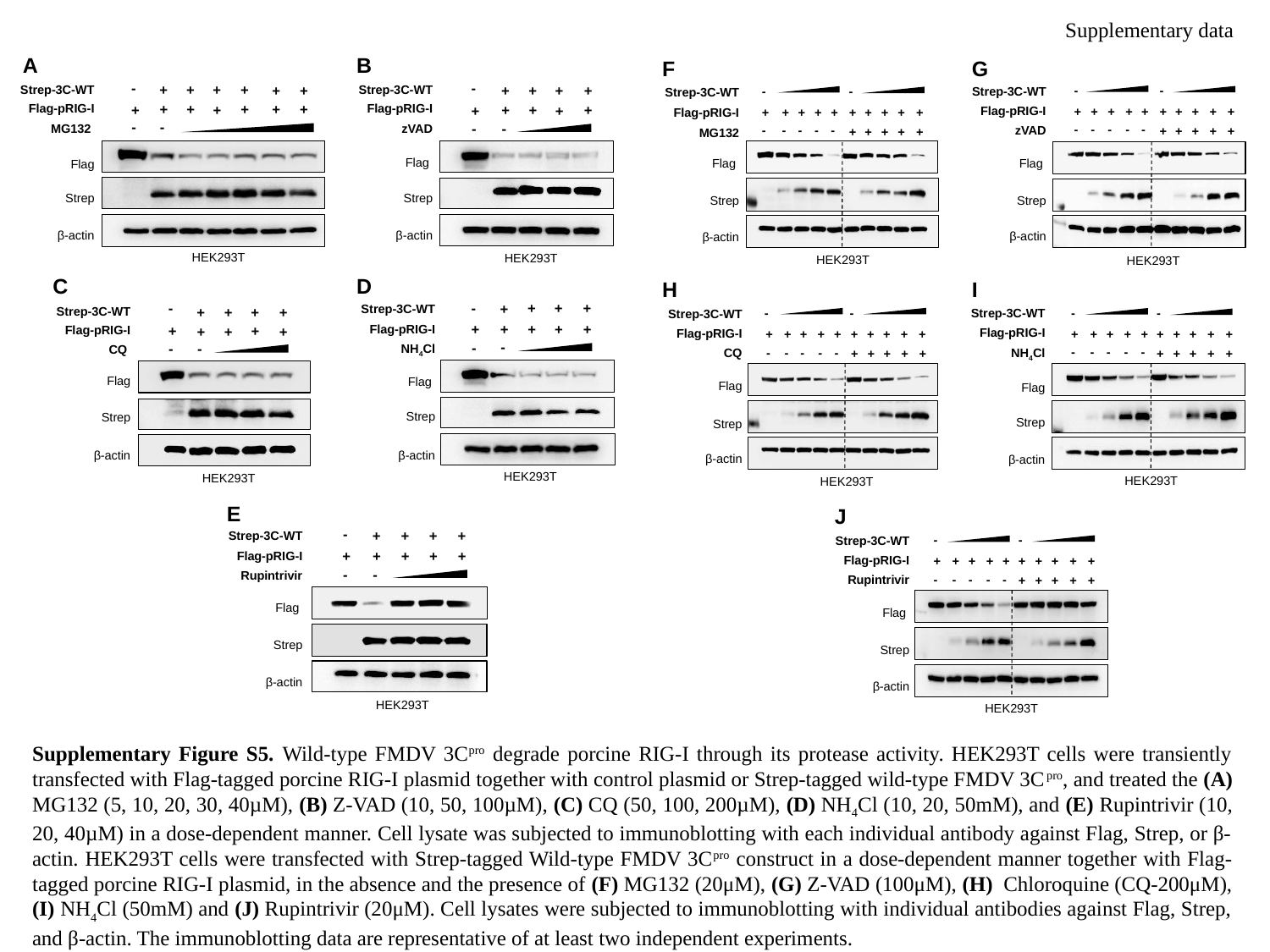

Supplementary data
A
B
F
G
-
+
+
+
+
+
+
Strep-3C-WT
Flag-pRIG-I
+
+
+
+
+
+
+
-
-
MG132
Flag
Strep
β-actin
HEK293T
-
Strep-3C-WT
+
+
+
+
Flag-pRIG-I
+
+
+
+
+
-
-
zVAD
Flag
Strep
β-actin
HEK293T
-
-
Strep-3C-WT
Flag-pRIG-I
+
+
+
+
+
+
+
+
+
+
-
-
-
-
-
zVAD
+
+
+
+
+
Flag
Strep
β-actin
HEK293T
-
-
Strep-3C-WT
Flag-pRIG-I
+
+
+
+
+
+
+
+
+
+
-
-
-
-
-
+
+
+
+
+
MG132
Flag
Strep
β-actin
HEK293T
C
D
H
I
-
+
+
+
+
Strep-3C-WT
Flag-pRIG-I
+
+
+
+
+
-
-
CQ
Flag
Strep
β-actin
HEK293T
-
+
+
+
+
Strep-3C-WT
+
+
+
+
+
Flag-pRIG-I
-
-
NH4Cl
Flag
Strep
β-actin
HEK293T
Strep-3C-WT
-
-
Flag-pRIG-I
+
+
+
+
+
+
+
+
+
+
-
-
-
-
-
NH4Cl
+
+
+
+
+
Flag
Strep
β-actin
HEK293T
-
-
Strep-3C-WT
Flag-pRIG-I
+
+
+
+
+
+
+
+
+
+
-
-
-
-
-
CQ
+
+
+
+
+
Flag
Strep
β-actin
HEK293T
E
J
-
+
+
+
+
Strep-3C-WT
+
+
+
+
+
Flag-pRIG-I
-
-
Rupintrivir
Flag
Strep
β-actin
HEK293T
-
-
Strep-3C-WT
Flag-pRIG-I
+
+
+
+
+
+
+
+
+
+
-
-
-
-
-
Rupintrivir
+
+
+
+
+
Flag
Strep
β-actin
HEK293T
Supplementary Figure S5. Wild-type FMDV 3Cpro degrade porcine RIG-I through its protease activity. HEK293T cells were transiently transfected with Flag-tagged porcine RIG-I plasmid together with control plasmid or Strep-tagged wild-type FMDV 3Cpro, and treated the (A) MG132 (5, 10, 20, 30, 40µM), (B) Z-VAD (10, 50, 100µM), (C) CQ (50, 100, 200µM), (D) NH4Cl (10, 20, 50mM), and (E) Rupintrivir (10, 20, 40µM) in a dose-dependent manner. Cell lysate was subjected to immunoblotting with each individual antibody against Flag, Strep, or β-actin. HEK293T cells were transfected with Strep-tagged Wild-type FMDV 3Cpro construct in a dose-dependent manner together with Flag-tagged porcine RIG-I plasmid, in the absence and the presence of (F) MG132 (20μM), (G) Z-VAD (100μM), (H) Chloroquine (CQ-200μM), (I) NH4Cl (50mM) and (J) Rupintrivir (20μM). Cell lysates were subjected to immunoblotting with individual antibodies against Flag, Strep, and β-actin. The immunoblotting data are representative of at least two independent experiments.

## Slide 6
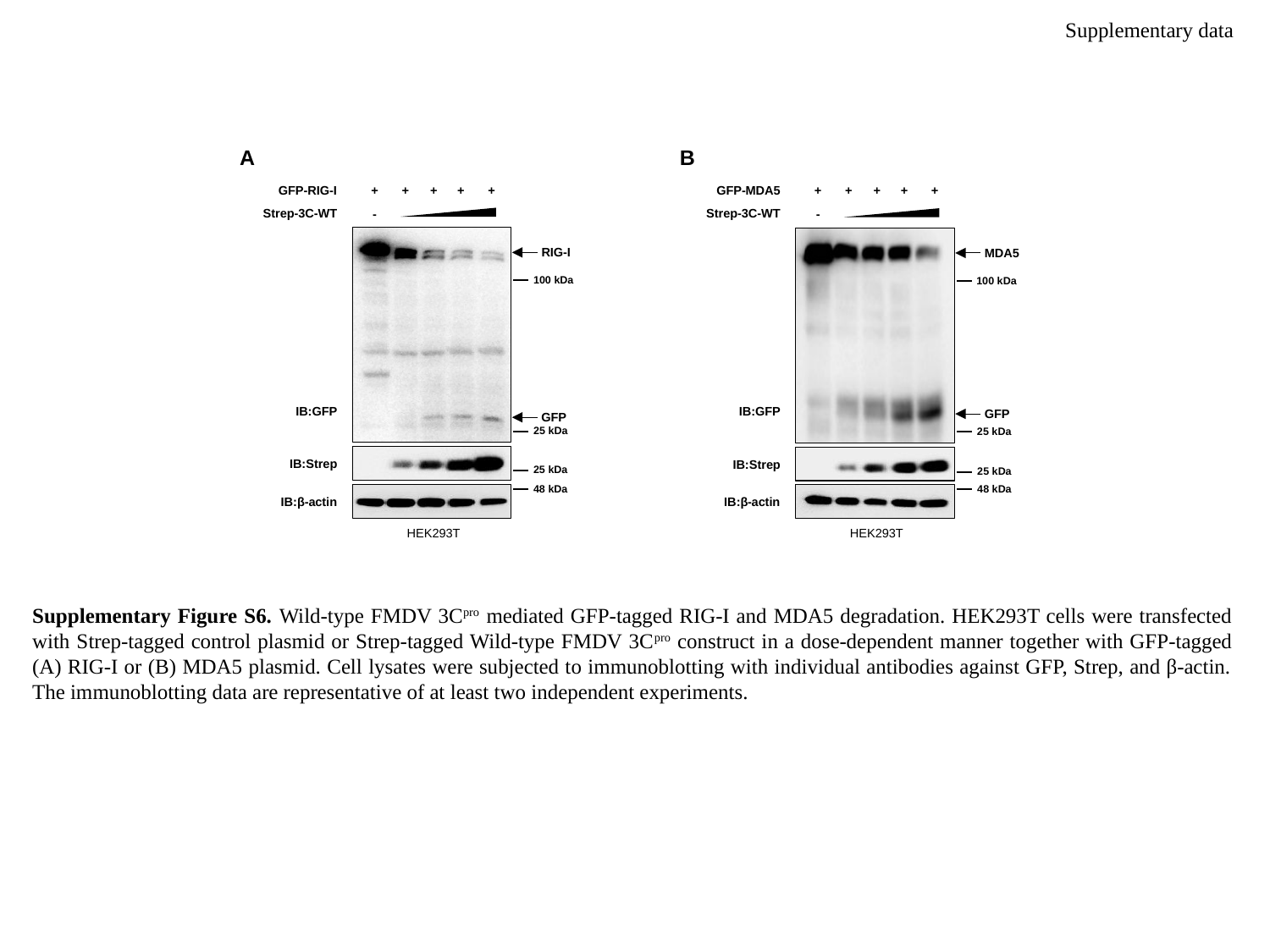

Supplementary data
A
B
+
GFP-RIG-I
+
+
+
+
Strep-3C-WT
-
RIG-I
100 kDa
IB:GFP
GFP
IB:Strep
IB:β-actin
HEK293T
25 kDa
25 kDa
48 kDa
+
GFP-MDA5
+
+
+
+
Strep-3C-WT
-
MDA5
100 kDa
IB:GFP
GFP
IB:Strep
IB:β-actin
HEK293T
25 kDa
25 kDa
48 kDa
Supplementary Figure S6. Wild-type FMDV 3Cpro mediated GFP-tagged RIG-I and MDA5 degradation. HEK293T cells were transfected with Strep-tagged control plasmid or Strep-tagged Wild-type FMDV 3Cpro construct in a dose-dependent manner together with GFP-tagged (A) RIG-I or (B) MDA5 plasmid. Cell lysates were subjected to immunoblotting with individual antibodies against GFP, Strep, and β-actin. The immunoblotting data are representative of at least two independent experiments.

## Slide 7
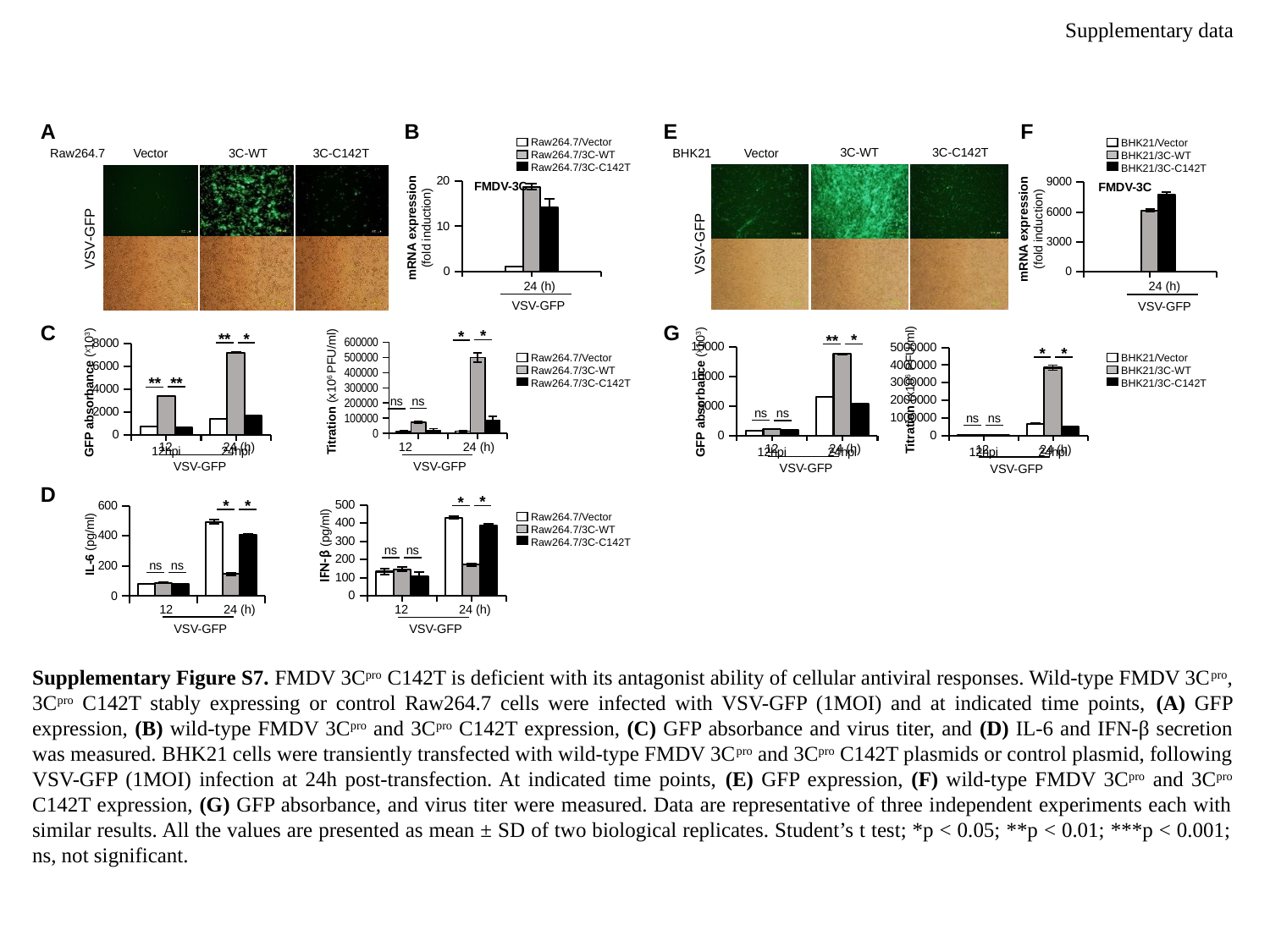

Supplementary data
A
B
E
F
Raw264.7/Vector
Raw264.7/3C-WT
Raw264.7/3C-C142T
BHK21/Vector
BHK21/3C-WT
BHK21/3C-C142T
3C-WT
3C-C142T
Vector
VSV-GFP
BHK21
Vector
3C-WT
3C-C142T
VSV-GFP
Raw264.7
FMDV-3C
### Chart
| Category | Control | 3C-WT | 3C - C142T |
|---|---|---|---|mRNA expression
(fold induction)
24 (h)
VSV-GFP
FMDV-3C
### Chart
| Category | Control | 3C-WT | 3C - C142T |
|---|---|---|---|mRNA expression
(fold induction)
24 (h)
VSV-GFP
C
G
*
*
### Chart
| Category | Control | 3C-WT | 3C-C142T mutant |
|---|---|---|---|
| 12hpi | 14500.0 | 73500.0 | 21000.0 |
| 24hpi | 16000.0 | 500000.0 | 86500.0 |Titration (x106 PFU/ml)
ns
ns
 12 24 (h)
VSV-GFP
### Chart
| Category | EV | 3C-WT | 3C - C142T mutant |
|---|---|---|---|
| 12hpi | 10250.0 | 51000.0 | 21900.0 |
| 24hpi | 665000.0 | 3850000.0 | 510000.0 |Titration (x106 PFU/ml)
 12 24 (h)
VSV-GFP
*
*
ns
ns
*
**
### Chart
| Category | Control | 3C-WT | 3C-C142T mutant |
|---|---|---|---|
| 12hpi | 746.954 | 3394.77 | 634.0609999999999 |
| 24hpi | 1395.925 | 7197.54 | 1712.2350000000001 |GFP absorbance (ˣ103)
 12 24 (h)
VSV-GFP
**
**
*
**
### Chart
| Category | Control | 3C - WT | 3C - C142T mutant |
|---|---|---|---|
| 12hpi | 799.2875 | 1094.24 | 904.1195 |
| 24hpi | 6490.71 | 13788.2 | 5296.785 |GFP absorbance (ˣ103)
 12 24 (h)
VSV-GFP
ns
ns
Raw264.7/Vector
Raw264.7/3C-WT
Raw264.7/3C-C142T
BHK21/Vector
BHK21/3C-WT
BHK21/3C-C142T
D
*
*
### Chart
| Category | Control | 3C-WT | 3C-C142T |
|---|---|---|---|
| 12hpi | 132.84300000000002 | 146.3586 | 108.70799999999998 |
| 24hpi | 430.18620000000004 | 170.4936 | 387.7086 |IFN-β (pg/ml)
ns
ns
 12 24 (h)
VSV-GFP
*
*
### Chart
| Category | Control | 3C-WT | 3C-C142T mutant |
|---|---|---|---|
| 12hpi | 78.49331 | 88.378885 | 77.92841999999999 |
| 24hpi | 493.12257 | 146.845 | 404.15239500000007 |IL-6 (pg/ml)
 12 24 (h)
VSV-GFP
ns
ns
Raw264.7/Vector
Raw264.7/3C-WT
Raw264.7/3C-C142T
Supplementary Figure S7. FMDV 3Cpro C142T is deficient with its antagonist ability of cellular antiviral responses. Wild-type FMDV 3Cpro, 3Cpro C142T stably expressing or control Raw264.7 cells were infected with VSV-GFP (1MOI) and at indicated time points, (A) GFP expression, (B) wild-type FMDV 3Cpro and 3Cpro C142T expression, (C) GFP absorbance and virus titer, and (D) IL-6 and IFN-β secretion was measured. BHK21 cells were transiently transfected with wild-type FMDV 3Cpro and 3Cpro C142T plasmids or control plasmid, following VSV-GFP (1MOI) infection at 24h post-transfection. At indicated time points, (E) GFP expression, (F) wild-type FMDV 3Cpro and 3Cpro C142T expression, (G) GFP absorbance, and virus titer were measured. Data are representative of three independent experiments each with similar results. All the values are presented as mean ± SD of two biological replicates. Student’s t test; *p < 0.05; **p < 0.01; ***p < 0.001; ns, not significant.

## Slide 8
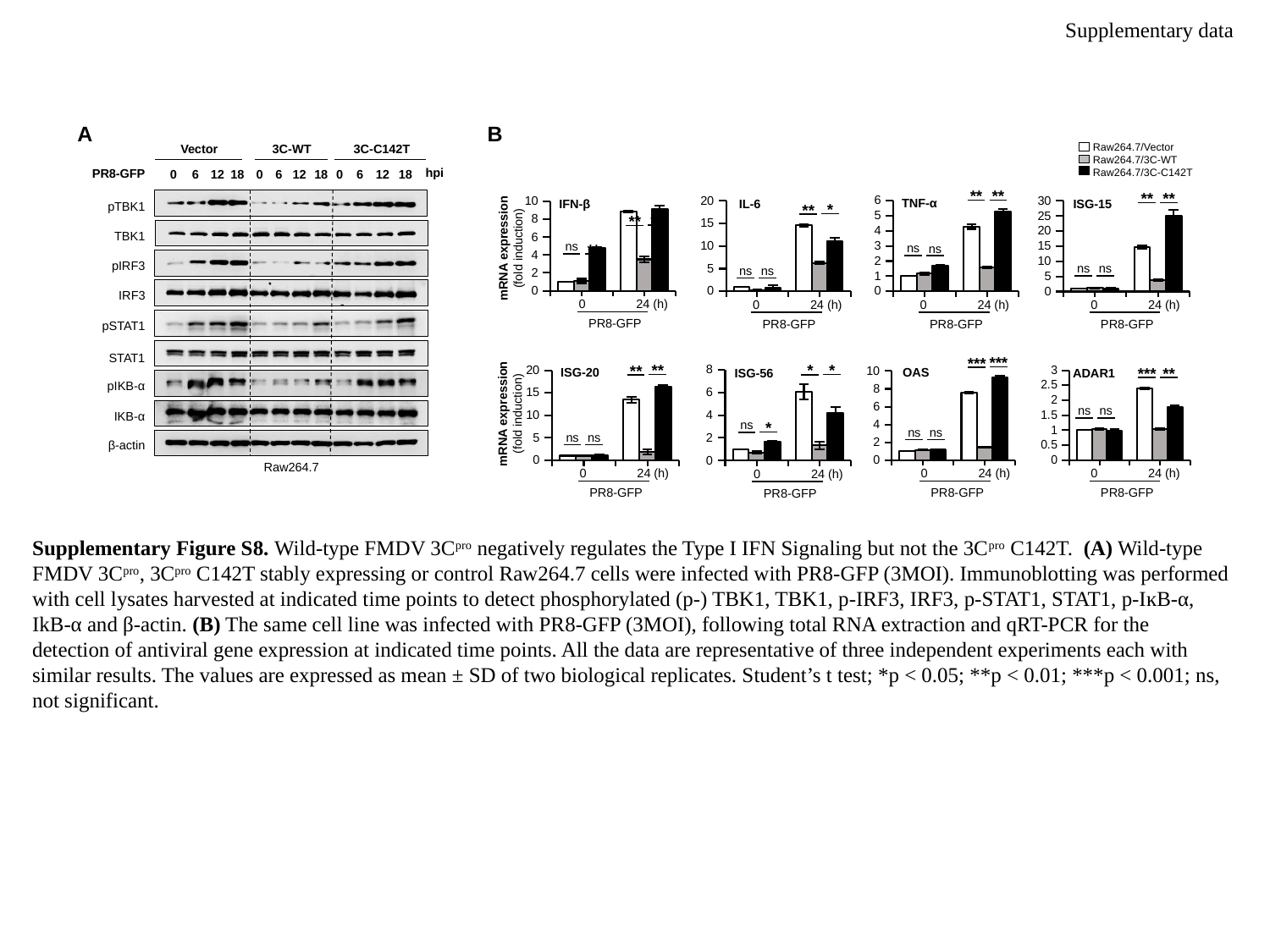

Supplementary data
A
B
Raw264.7/Vector
Raw264.7/3C-WT
Raw264.7/3C-C142T
3C-WT
3C-C142T
Vector
hpi
PR8-GFP
18
0
6
18
12
0
18
0
6
6
12
12
pTBK1
TBK1
pIRF3
IRF3
pSTAT1
STAT1
pIKB-α
IKB-α
β-actin
Raw264.7
### Chart
| Category | Con. | 3C-WT | 3C-C142T |
|---|---|---|---|
| 0h | 1.0 | 1.165 | 1.7 |
| 24h | 4.275 | 1.575 | 5.295 |TNF-α
 0 24 (h)
PR8-GFP
**
**
ns
ns
**
**
### Chart
| Category | Con. | 3C-WT | 3C-C142T |
|---|---|---|---|
| 0h | 1.0 | 1.165 | 1.075 |
| 24h | 14.735 | 3.745 | 25.095 |ISG-15
 0 24 (h)
PR8-GFP
ns
ns
IFN-β
### Chart
| Category | Con. | 3C-WT | 3C-C142T |
|---|---|---|---|
| 0h | 1.0 | 1.095 | 4.76 |
| 24h | 8.895 | 3.51 | 9.145 |mRNA expression
(fold induction)
 0 24 (h)
PR8-GFP
**
**
ns
**
### Chart
| Category | Con. | 3C-WT | 3C-C142T |
|---|---|---|---|
| 0h | 1.0 | 0.23500000000000001 | 0.735 |
| 24h | 14.58 | 6.285 | 11.125 |IL-6
 0 24 (h)
PR8-GFP
*
**
ns
ns
***
***
### Chart
| Category | Con. | 3C-WT | 3C-C142T |
|---|---|---|---|
| 0h | 1.0 | 1.145 | 1.16 |
| 24h | 7.545 | 1.45 | 9.32 |OAS
 0 24 (h)
PR8-GFP
ns
ns
### Chart
| Category | Con. | 3C-WT | 3C-C142T |
|---|---|---|---|
| 12h | 1.0 | 1.0 | 1.12 |
| 24h | 13.504999999999999 | 1.83 | 16.275 |ISG-20
mRNA expression
(fold induction)
 0 24 (h)
PR8-GFP
**
**
ns
ns
### Chart
| Category | Con. | 3C-WT | 3C-C142T |
|---|---|---|---|
| 12h | 1.0 | 0.72 | 1.625 |
| 24h | 6.0600000000000005 | 1.355 | 4.1899999999999995 |ISG-56
 0 24 (h)
PR8-GFP
*
*
ns
*
**
***
ADAR1
### Chart
| Category | Con. | 3C-WT | 3C-C142T |
|---|---|---|---|
| 0h | 1.0 | 1.045 | 0.9850000000000001 |
| 24h | 2.3899999999999997 | 1.045 | 1.7850000000000001 | 0 24 (h)
PR8-GFP
ns
ns
Supplementary Figure S8. Wild-type FMDV 3Cpro negatively regulates the Type I IFN Signaling but not the 3Cpro C142T. (A) Wild-type FMDV 3Cpro, 3Cpro C142T stably expressing or control Raw264.7 cells were infected with PR8-GFP (3MOI). Immunoblotting was performed with cell lysates harvested at indicated time points to detect phosphorylated (p-) TBK1, TBK1, p-IRF3, IRF3, p-STAT1, STAT1, p-IĸB-α, IkB-α and β-actin. (B) The same cell line was infected with PR8-GFP (3MOI), following total RNA extraction and qRT-PCR for the detection of antiviral gene expression at indicated time points. All the data are representative of three independent experiments each with similar results. The values are expressed as mean ± SD of two biological replicates. Student’s t test; *p < 0.05; **p < 0.01; ***p < 0.001; ns, not significant.
